# Volume–outcome relationship in anatomical and non-anatomical liver resections: a rapid systematic review

**DOI:** 10.1186/s12876-025-04490-x

**Published:** 2026-02-25

**Authors:** Alessandro Campione, Julian Modrow, Helene Eckhardt, Cinara Paul, Ulrike Nimptsch, Cornelia Henschke

**Affiliations:** 1 Department of Health Care Management, Technische Universität Berlin, H80, Straße des 17. Juni 135, 10623 Berlin, Germany; 2 Institute of General Practice and Interprofessional Care, University Hospital Tübingen, Faculty of Medicine, Eberhard Karls Universität Tübingen, Tübingen, Germany

**Keywords:** Liver resection, Surgical volume, Hospital performance, Surgeon experience, Postoperative complications, Mortality outcomes, Health services research, Quality of care

## Abstract

**Background:**

Despite considerable advancements in recent decades, mortality and complications following liver resection remain high. The volume-outcome relationship has been the subject of extensive research and offers relevant potential for improvement of surgical outcomes. This review aims to examine the impact of hospital and surgeon volume on patient-relevant outcomes in liver resections and synthesize the available evidence.

**Methods:**

A rapid systematic literature review was conducted, searching CENTRAL, Embase, PubMed, and study registries for articles published from 2000 to 2023. Eligible studies investigated the association between hospital or surgeon volume and patient-relevant outcomes in anatomical and non-anatomical liver resections. Study quality was assessed using the ISPOR and ROBINS-E checklists and reported alongside the results. The review protocol registered with PROSPERO (CRD42023398566).

**Results:**

The search yielded 3287 records, of which 38 publications met the inclusion criteria. All included studies were retrospective observational studies. A higher surgical volume was associated with improved patient-relevant outcomes, such as reduced mortality following both anatomical and non-anatomical liver resections and lower rates of postoperative complications. However, the results indicate that the impact of hospital or surgeon volume is limited and likely depends on the respective outcome parameter. A considerable gap remains with respect to long-term outcomes and quality of life, and studies investigating surgeon volume are scarce.

**Conclusion:**

The findings provide evidence supporting a positive association between higher hospital volume and improved patient-relevant outcomes in liver resection. However, surgeon volume remains underexplored and the evidence from subgroups indicates that the impact of hospital or surgeon volume likely depends on study quality, procedure type, volume thresholds, and respective outcome parameters. Patient care could benefit from further research on long-term outcomes as well as quality of life, for which the current evidence is scarce.

**Supplementary Information:**

The online version contains supplementary material available at 10.1186/s12876-025-04490-x.

## Introduction

Resection of the liver has become the standard treatment for liver cancer [1]. The most frequent surgical indications include metastatic diseases and malignant neoplasms of the liver [2, 3]. Liver cancer represents a significant global health burden, with increasing incidence rates, representing a major cause of cancer-related mortality worldwide [4, 5]. Since the year 2000, the field of liver surgery has achieved significant advancements in obtaining favorable operative outcomes, mostly related to technical innovations [6, 7]. Nevertheless, liver resections still exhibit high mortality, morbidity, and complications rates [8–10], additionally contributing to significantly increased healthcare costs [11]. The volume-outcome relationship has been the subject of extensive research, suggesting that higher hospital or surgeon volumes are associated with improved postoperative outcomes [12] and lower costs [13, 14]. Several studies have demonstrated this relationship between hospital volume and outcomes in multiple surgical disciplines [15], such as colorectal cancer surgery [16], and gynecologic oncology [17]. In pancreatic surgery, higher surgeon volumesmes have similarly been linked to improved outcomes [18, 19]. The underlying mechanism is thought to involve enhanced technical proficiency and more effective organizational structures in high-volume centers, consistent with the “practice makes perfect” paradigm [20, 21]. Accordingly, several European countries have implemented minimum-volume standards for liver resections [22]. In Switzerland and the Netherlands, hospitals are required to perform at least 20 liver resections per, while 10 per year are required in Austria [23, 24]. Other countries are yet to establish such standards, and instead rely on alternative regulatory instruments, such as voluntary certification programs in Germany [25, 26] or centralized service planning based on catchment areas in Denmark [22].

Although primary studies have examined the volume-outcome relationship in liver surgery [27–30], there is a limited number of up-to-date systematic reviews analyzing different outcomes, surgery types and surgery extents. For instance, aFor instance, a review published in 2020 focused on hepatobiliary surgeries only [31]. Two earlier reviews found evidence supporting a volume-outcome association for short-term mortality [32, 33] but inconclusive evidence regarding length of stay, morbidity, and long-term survival, partly due to a small number of relevant studies. Both reviews were based primarily on studies published before 2010, and the meta-analysis published in 2013 included only studies addressing hospital-volume [33]. Other works have explored the combined effect of surgeon and hospital-volume [34] or focused solely on major liver resections for perihilar cholangiocarcinoma (PHC) [35]. A recent rapid review including ten studies found significant volume-mortality associations but limited its scope to major and anatomic resections [36], whereas a 2024 meta-analysis focused solely on postoperative short-term mortality in major or minor liver resections [37]. Consequently, further research is warranted to synthesize the available evidence on the volume-outcome relationship in liver resection, identify existing evidence gaps, and generate actionable insights for healthcare policy. This review addresses these objectives across all types of liver resection procedures and adds an underexplored stratification by anatomical or non-anatomical liver resections, which differ in complexity and may therefore exhibit distinct volume effects.

## Methods

A rapid review was conducted, aiming to inform clinicians and health policy decision makers. This type of review was chosen against the policy backdrop of hospital reform in Germany, which intends to base future hospital planning on defined service groups, including liver resection [38, 39]. This rapid systematic review was registered with PROSPERO (CRD42023398566) and followed the Cochrane Handbook for Systematic Reviews of Interventions and the InterimGuidance of the Cochrane Rapid Reviews Methods Group [40]. Furthermore, the Preferred Reporting Items for Systematic Review and Meta-Analyses (PRISMA) statement was consulted to guide reporting [41].

### Eligibility criteria

The following eligibility criteria following PICOS [42] were applied:

#### Population

Adult patients (≥ 18 years old) who underwent either anatomic or non-anatomic major or minor liver resection for malignant or non-malignant diseases of the cirrhotic or non-cirrhotic liver.

#### Intervention/Exposure

Surgical procedures included anatomic or non-anatomic major or minor liver resections. These may include any combination of liver segments and open or minimally invasive laparoscopic surgical (MILS) approaches. This review excluded (partial) liver transplantations or liver resections for transplantation in healthy individuals.

#### Context

The aim was to evaluate the relationship between volume and outcome at surgeon and hospital level. Volume was defined as the number of liver resections performed in a hospital or by a surgeon within a given period. Only studies that incorporated a comparison between low and high-volume hospitals (LVH; HVH) or a comparison between low and high-volume surgeons (LVS; HVS) were included. Additionally, volume analyzed as a continuous variable was included. Studies analyzing data from a single hospital or surgeon were excluded.

#### Outcomes

Studies that examined at least one of the following outcomes: (1) Mortality (any time frame, including in-hospital mortality, short-term mortality (30- or 90-day), intermediate or long-term mortality (1 to 5 years) or failure to rescue (FTR)), (2) Morbidity (perioperative morbidity, complications or disease-related morbidity), (3) Length of stay (LOS) in: hospital or intensive care unit (ICU), (4) Health-related quality of life (HRQoL) assessed using validated measurement instruments.

If the impact of volume on other relevant outcomes was investigated, these were included. For instance, studies investigating textbook outcomes (TOs) were considered. TO are composite outcome indicators that represent the optimal course following surgery [43].

#### Study design

Primary studies, such as randomized controlled trials (RCTs), observational, or intervention studies or trials (retrospective and prospective cohort studies) published between 2000 and 2023 in peer-reviewed journals and registry entries were eligible. Systematic reviews were used to identify additional relevant studies from the reference lists but were not included for synthesis themselves. Multi-publications were excluded unless they reported different outcome parameters. Inclusion was limited to studies published in English or German with full-text availability.

#### Adjustments to the inclusion criteria

Following full-text screening, studies published prior to 2010 were excluded. This year was chosen as it coincides with the uptake of new surgical technologies in liver resection (laparoscopic surgery) [44], a stabilization and plateau in mortality of hepatocellular carcinoma (HCC) [45] as well as an update to the American Association for the Study of Liver Diseases (AASLD), which includes relevant changes for the treatment of cancer of the liver [46]. Most of the studies included both anatomical and non-anatomical liver resections (commonly referred to as partial hepatectomies or wedge resections). Therefore, studies were considered eligible for inclusion if the procedure type could be determined with certainty.

### Information sources

Searches were conducted in PubMed (via PubMed), Embase (via Ovid), and CENTRAL (via Cochrane Library) in January 2023. Furthermore, three clinical trial registries were searched in March 2023: International Clinical Trials Registry Platform (ICTRP), ClinicalTrials.gov and German Clinical Study Register (DRKS). A manual search of the reference lists from relevant identified systematic reviews was performed to identify additional studies. Following initial scoping searches in PubMed, a draft search strategy was developed in accordance with the Peer Review of Electronic Search Strategies (PRESS) guidelines [47]. The initial search strategy was subsequently adapted for Embase and the Cochrane Library and tested in each database. Each search string included both free-text terms and database-specific subject headings, such as Medical Subject Headings (MeSH) in PubMed, and Emtree terms in Embase. The search strings are provided in Appendix 1 in Supplementary Material 1. In addition, registry searches were conducted in accordance with methodological recommendations [48]. Automated update alerts were activated in all databases, and the final search update was conducted on March 6, 2023. Grey literature was not included.

### Data management and selection of relevant studies

Titles and abstracts were deduplicated using manual and automatic methods (Endnote 9.1). Initially, a pilot test involving 40 PubMed records was performed to evaluate the predefined eligibility criteria and ensure consistency. Two reviewers (JM, AC) independently screened a random 20% sample of titles and abstracts to calibrate the screening process, aiming for an agreement of ≥90%. Upon reaching the predefined threshold, one reviewer (JM) proceeded to screen the remaining 80% of the records. Prior to the full-text screening, a second pilot test was performed by the same researchers on a random 20% sample of the full text records. Subsequently, one reviewer (JM) assessed the full-texts of all studies included after title-abstract screening using Zotero, while a second reviewer independently screened all excluded articles (AC). In cases where full-texts or essential eligibility information were unavailable, the corresponding authors were contacted. If no response was received, the respective titles were excluded. Any disagreements during the screening process were resolved through discussion or, if necessary, consultation with additional reviewers (HE, CH).

### Data extraction

A standardized Excel spreadsheet was developed and pilot-tested by both reviewers using three randomly selected studies. One reviewer extracted the relevant items from included titles (JM), which were verified by a second reviewer (AC). Extracted items included study and patient characteristics, volume classifications, statistical methods, and reported outcomes. Outcome data were collected in both unadjusted and adjusted forms where available. If relevant data were not reported or only available graphically, inquiries were made to the authors via e-mail. If no response was received within 14 days, the respective studies were excluded from the analysis.

### Data synthesis and risk of bias assessment

The study characteristics and results were presented in tabular form and narratively synthesized. A meta-analysis was not performed due to substantial heterogeneity in study design, populations, outcome definitions, and analytical approaches, as well as variability in study quality, which made statistical pooling inappropriate. Where information was missing but could be calculated from reported data, values were calculated and clearly indicated. Results were reported separately for each outcome and stratified by volume and resection type. If results were not reported distinctly for subgroups, they were reported as intersections of the subgroups. To ensure completeness, both adjusted and unadjusted outcomes were included in the results tables. However, synthesis only considered adjusted results.

The quality of included studies was assessed using the checklist for retroactive database studies provided by the International Society for Pharmacoeconomics and Outcome Research (ISPOR) [49]. The checklist contains 27 items that focus on challenges specific to retrospective databases, disease registries, and national survey data. Each item was assessed using the categories “Yes”, “Partially”, “No”, or “Not Applicable” (NA). As the checklist does not yield an overall summary score, the number of responses in each category was reported per study alongside the results. Furthermore, domain 7 (Risk of bias in the selection of the reported results) of the “Risk of Bias In Non-randomized Studies – of Exposure” (ROBINS-E) tool was applied to further assess the quality of outcome reporting [50]. This domain contains five items with response options “Yes”, “Probably Yes”; “Probably No”; “No” or “No Information”. Question 7.1 (Was the reporting of the result in accordance with an available, pre-determined analysis plan?) was rated as “No Information” for all included studies, as no analysis plans were available. 

To assess overall quality, the ISPOR checklist was supplemented with domain 7 from the ROBINS-E tool. A composite quality score was calculated by assigning numerical values to selected response categories from both instruments. Specifically, the total score per study was derived by summing the number of “Yes” ratings on the ISPOR checklist (1 point each), adding 0.5 points for each “Partially” rating, and adding 2 points for each “Low Risk” judgment in ROBINS-E (domain 7), according to the following formula:

$$\begin{aligned} \:Quality\:Score\:\\&={X}_{ISPOR\:"Yes"}\\&+0.5*{Y}_{ISPOR\:"\mathrm{Partially"}}\\&+2*1\!\!1_{ROBINS\:"Low\:Risk"} \end{aligned}$$

In each stratum, studies analyzing mortality, morbidity, LOS and TOs were ranked according to their quality score.

## Results

### Study selection process

**Fig. 1:**
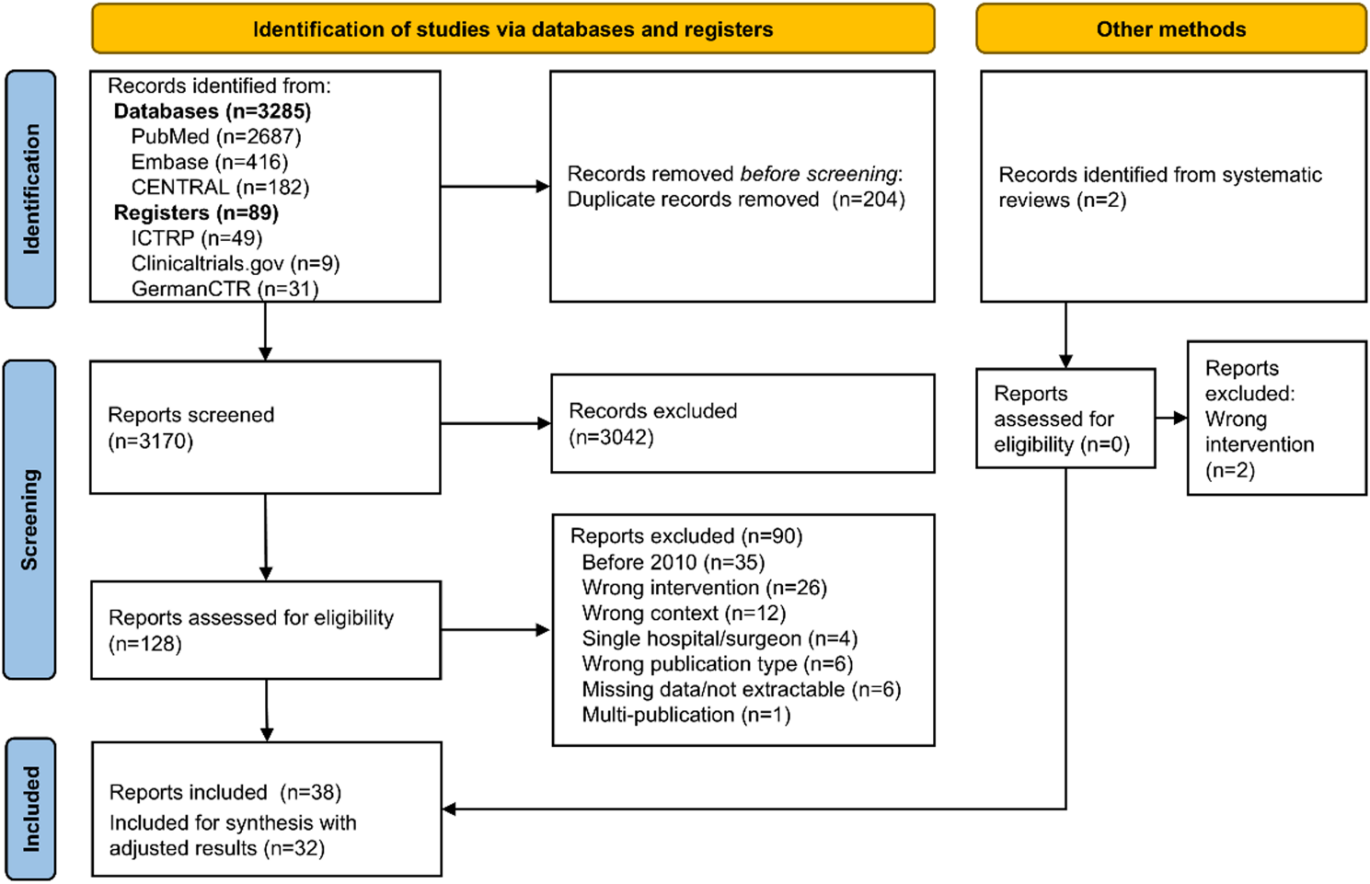
Study selection process

A total of 3376 records were identified. Among the two records identified through reference screening of systematic reviews, none met the inclusion criteria. Consequently, 128 full texts were assessed for eligibility. Ultimately, 38 reports met the inclusion criteria and 32 reports with adjusted results were included in the synthesis. The selection process is illustrated in Figure 1.

The primary reasons for exclusion were intervention and study context. Specifically, studies were excluded if they analyzed non-hepatic surgeries, such as pancreatic resections or ablations, without reporting separate results for hepatic interventions. Context-related exclusions were applied to studies that lacked clear or consistent definitions of surgical volume. A complete list of excluded studies at full-text level is provided in Appendix 2 in Supplementary Material 1.

Further adjustments for multi-publications were made to avoid duplication of data while preserving relevant outcome information. In two publications, similar morbidity outcomes were analyzed using the same dataset for morbidity [14, 51]. The more recent one, which additionally investigated FTR was included [51]. Similarly, two Taiwanese publications using the same data and identical inclusion criteria but differing in analytical methods and outcomes were treated as a single source and referred to as Chiu et al. (2015) [52, 53]. In contrast, two multi-publications by similar authors were included as separate studies due to differing time frames and outcomes [54, 55]. Three Japanese studies from closely associated authors relied on the same database and examined the same procedures but were not merged due to different inclusion criteria [56–58]. Finally, two Dutch studies used similar clinical datasets and investigated the same outcomes over the same period, however, the 2021 study additionally included data from international high volume centers [59, 60] and was therefore retained separately. 

Although a German study published in 2019 initially appeared not to meet the inclusion criteria − due to the use of procedure codes for incision, local excision of the liver, or local tumor destruction − it was ultimately included in the synthesis [9]. This decision was based on direct communication with the authors, who confirmed that the study explicitly included anatomical liver resections for the purpose of volume-outcome assessment. In addition, several other studies utilized data derived from the same databases with overlapping study periods. These studies were not excluded, as they differed substantially in study populations, methodological approaches, or outcome definitions, thereby justifying their separate inclusion in the synthesis.

### Study and patient characteristics

The collective patient population encompassed a total of 552155 patients, with the largest study analyzing a cohort of 96107 patients [51]. A majority of 20 studies were conducted in the US, followed by eleven studies from European countries, six from Asia, and one from Canada. The included studies were published between 2010 and 2023, with data collection periods ranging from 1995 [61] to 2019 [62]. All studies were based on retrospective institutional or population-based cohorts; no randomized controlled trials (RCTs) were identified. In total, 14 distinct data sources were used. In ten studies, procedure codes specifying the types of liver resections were not reported [56, 59, 60, 62–68]. To capture the extent of liver resections performed, available details on resection types were extracted and presented in Appendix 3 in Supplementary Material 1. Data on the extent of liver resection were not reported in four studies [52, 69–71]. Several studies focused specifically on anatomical resections [2, 9, 64, 67, 72–75], of which three investigated patient-relevant outcomes following lobectomies [72, 75, 74]. Three studies included a comprehensive range of anatomical resection types [2, 9, 73], such as single segmentectomies, bisegmentectomies, trisegmentectomies, and left- or right hepatectomies. In total, seven studies focused exclusively on patients who underwent anatomical liver resections, whereas the remaining 31 studies included patients undergoing either anatomical or non-anatomical resections. Hospital-volume was investigated in 29 studies, while four studies used surgeon-volume as the independent variable [61, 70, 76, 77]. Five studies examined the effect of both hospital and surgeon-volume. Most studies examined volume as categorical variables or statistical quantiles. Four studies [64, 70, 74, 77, 78] also expressed volume as a continuous variable, with two studies relying solely on continuous volume analyses [70, 74] (Table 1). A small number of studies did not adequately report data on volume-related data, such as the number of surgeries or the number of patients per volume category. Volume definitions are shown in Appendix 4 in Supplementary Material 1.

**Table 1:** Characteristics of included studies

First author and year of publication	Funding	Country (region)	Study period	Study type Data source	Intervention^1^	Unit	Units (*N*)	Indication^1^	Patients (*N*)	% Female	Age (years)
Ardito 2020 [65]	-	IT	2010-2012	Retrospective HE.RC.O.LE.S.	A; NA	H	18	HCC	1935	24.5	Median 71
Beal 2019 [78]	-	USA & PR	2004-2015	Retrospective NCDB	A; NA	H	-	HCC	12,226	70.5	Median 64
Buettner 2016 [80]	None	US	2000-2009	Retrospective NIS	A; NA	H	408	Primary/secondary liver malign	5075	44.8	Median 62
						S	1099				
Chang 2014 [63]	None	TW	2002-2006	Retrospective NHI	A; NA	H/S	-	HCC, ICC, benign disease, secondary malign.	13,159	35.6	Mean 55.06 Mean 58
Chapman 2017 [82]	Non-profit	US & PR	1998-2011	Retrospective NCDB	A; NA	H	1060	HCC, benign disease	12,757	32.1^a^	<50: 1846 50–79: 9925≥ 80: 946
Chiu 2015 [52] (Lu 2014 [53])	None	TW	1998-2009	Retrospective NHI	A; NA	H/S	-	Malign. of liver or intrahepatic bile ducts	23,107	26.4	Median 60
Dhar 2019 [72]	None	US	2011-2014	Retrospective UHC	A	H/S	-	-	6476	49.9	Median 57
Diggs 2021 [67]	None	US & PR	2006-2015	Retrospective NCDB	A	H	607	HCC, iCCA	4263	33	<65: 1978 ≥65: 2285^α^
Eguia 2021 [79]	Non-profit	US (FL, MD, NY, NC, WA)	2010-2014	Retrospective HCUP, SID	A; NA	H	261	Primary/secondary malign, benign, other	10,239	51.8^a^	OS: Mean 59
El Amrani 2019 [69]	-	FR	2012-2017	Retrospective PMSI	A; NA	H	715	Malign.	28,763	34.7	<60: 8701; 60–79: 18,266^a^ ˃80: 1796
Endo 2023 [86]	-	US & PR	2010-2018	Retrospective NCDB	A; NA	H	432	HCC	3268	29.4	Median 65
Farges 2012 [88]	Non-profit	FR	2007-2010	Retrospective PMSI	A; NA	H	533	Primary/secondary malign., benign, parasitic or liver abscesses, other	22,275	45.7	Mean 61.1
Filmann 2019 [9]	-	DE	2010-2015	Retrospective DRG statistics	A	H	-	CRLM, ECC, GCC, HCC, ICC	40,034	-	<50: 15,704 50–69: 49,966^a^ >60: 44,662
Gani 2016 [55]	-	US	2001-2012	Retrospective NIS	A; NA	H	2207	Primary/secondary malign, cirrhosis	27,813	55.9	Median 61
Gani 2017 [54]	-	US	2001-2011	Retrospective NIS	A; NA	H	1573	Primary/secondary malign.	14,296	46.3	Median 60
Görgec 2021^b^ [59]	Profit, Non-profit	NO, UK, IT, NL	2011-2016	Retrospective patient files	A; NA	H	23	CCA, CRLM, HCC, benign, other	1540^e^ 885^f^	LV: 48.4 HV: 48.0	LV: Median 64 High: Median 66
van der Poel 2019 [60]	-	NL	2011-2016	Retrospective patient files	A; NA	H	20	CCA, CRLM, HCC, other	916	LV 47.0 HV: 38.0	LV: Median 64 HV: Median 66
Hashimoto 2017 [70]	Non-profit	US (NY)	2000-2014	Retrospective SPARC	A; NA	S	909	-	13,467	50	-
Hoerger 2023 [89]	-	US & PR	2004-2017	Retrospective NCDB	A; NA	H	446	HCC, ICC	17,833	33.7	LV: Median 66 HV: Median 64
Hunger 2019 [73]	-	DE	2011-2015	Retrospective DRG	A	H	^d^	Secondary malign. of liver/intrahepatic bile ducts and colon	5900	39.9	<49: 487 50–79: 5044^a^ >80: 369
Idrees 2018 [51]	-	US	2002-2011	Retrospective NIS	A; NA	H	-	-	96,107	-	Median 59
Kohn 2010 [74]	-	US	1998-2006	Retrospective NIS	A	H	1045		5298	-	-
Krautz 2020 [2]	Non-profit	DE	2009-2015	Retrospective DRG	A;	H	^d^	Primary/secondary malign, benign, other	31,114	^d^	^d^
Lee 2019 [87]	Non-profit	US & PR	2004-2014	Retrospective NCDB	A; NA	H	308	ICC	2256	53.6^a^	<65: 1144^a^≥65: 1112^α^
Magnin 2023 [62]	Non-profit	FR	2011-2019	Retrospective PMSI	A; NA	H	336	Malign, benign	39,286	39.8^a^	Mean 63.5
McColl 2013 [61]	Non-profit	CA (CGY, EDM)	1995-2004	Retrospective CIHI	A; NA	S	67	Primary/secondary malign, other	1033	53.6^a^	CGY: Median 57 EDM: Median 60
Miura 2016 [64]	-	JP	2011-2012	Retrospective NCD	A; NA	H	1047	GCC, HCC, ICC, pCCA, secondary malign, other	14,970	29.9^a^	Mean 67
Munir 2023 [90]	-	US & PR	2004-2018	Retrospective NCDB	A; NA	H	-	ICC	5359	53.7^a^	<64: 2376 64–75: 2184^a^ >75: 799
Okinaga 2018 [56]	Non-profit	JP	2007-2012^c^	Retrospective DPC	A; NA	H	952	HCC	27,094	28.7	≤59: 5099 60–84: 21,625^a^≥85: 370
Sato 2012 [57]	Non-profit	JP	2007-2008^c^	Retrospective DPC	A; NA	H	808	HCC	5270	29.3	Mean 67.7
Yasunaga 2012 [58]	Non-profit	JP	2007-2009^c^	Retrospective DPC	A; NA	H	855	Primary/secondary malign., others	18,046	31.7	Mean 66.8
Sahara 2020 [76]	None	US	2013-2015	Retrospective SAF	A; NA	S	3403	Malign., benign	7169	49	Median 72
Shaw 2013 [71]	-	US	2007-2010	Retrospective UHC	A; NA	H	50	Primary/secondary malign., benign	8692	54.2^a^	GS: median 57 SS: median 57
						S	730				
Siegel 2021 [68]	None	US & PR	2004-2014	Retrospective NCDB	A; NA	H	-	HCC	6860	32.2	Mean 62
Spolverato 2014 [83]	-	US	2000-2010	Retrospective NIS	A; NA	H	195	Primary/secondary malign	9874	43.8	Median 61
Sutton 2016 [75]	-	US	2009-2011	Retrospective UHC	A	H	109^a^	-	4163^a^	51	Median 58
Tsilimigras 2021^g^ [77]	None	US	2013-2017	Retrospective SAF	A; NA	S	557	Malign	13,222	49.1	Median 71
Viganò 2020^h^ [66]	-	IT	2014-2018	Retrospective I GO MILS	A; NA	H	46	CRLM, HCC, MFCC, benign, other	2225	42.5^a^	>70: 853

*A* anatomical, *CA* Canada, *CCAM*, *CCI* Canadian classification of Health Interventions, *CGY* Calgary, *CIHI* Canadian Institute for Health Information database, *DE* Germany, *DPC* Diagnosis Procedure Combination database, *EDM* Edmonton, *FR* France, *GS* General surgeon, *ICC* intrahepatic cholangiocarcinoma, *H* hospital, *HCC* hepatocellular carcinoma, *HCUP*-*SID* Healthcare Cost and Utilization Project and State Inpatient Databases *HE.RC.O.LE.S.* Hepatocarcinoma Recurrence on the Liver Study Group, *HV* High volume, *I GO MILS* Italian Group of Minimally Invasive Liver Surgery registry, *JP* Japan, *LS* Laparoscopic surgery, *LV* Low volume, *MBDSHD* minimum basic data set of hospital discharge, *NCD* National Clinical Database, *NCDB* National Cancer Database, *NHI* National Health Insurance Research Database, *NIS* Nationwide Inpatient Sample, *OS* Open surgery, PMSI Programme de Médicalisation des Systémes d’Information, *PR* Puerto Rico, *SAF* Medicare Inpatient and Outpatient Standard Analytic Files, *SPARC* New York Statewide Planning and Research Cooperative System inpatient database, *SS* Specialist surgeon, STORE Standards for Oncology Registry Entry, *S* Surgeon, *TW* Taiwan, *UHC* University Health System Consortium, *UK* United Kingdom, *US* United States

^a^ self-calculated

^b^ data from three high-volume centers in Oslo (NO), Southhampton (UK), Milan (IT), Amsterdam (NL) and 20 high-volume centers in NL; authors received an educational grant from Johnson & Johnson Medical B.V. and Integra LifeSciences

^c^ July-December

^d^ reported/year, available from digital appendix

^e^ international hospitals (NO, UK, IT, NL)

^f^ national hospitals (NL)

^g^ Study included 13,222 patients: 13,100 open surgery, 1112 MILS

^h^ November 2014 – May 2018

^1^ Available from appendix: surgery type, procedure codes, extent of surgery, diagnosis code

### Outcomes, including quality appraisal

#### Textbook outcomes and hospital-volume

TOs were analyzed at hospital-volume level in only two studies [90, 86] (Table 2). Both included anatomical and non-anatomical resections and reported significantly higher TO rates in HVHs. One study defined HVHs as ≥50 minimally invasive hepatectomies (MIH) over 6-years [86]. The other study categorized hospitals into three volume groups, observing the strongest effect when comparing HVHs (>7 cases/year) with LVHs (<3 cases/year) [90]. Both studies were based on comparable cohorts from the US National Cancer Database (NCDB).

**Table 2:** Textbook-outcomes (hospital-volume level)

First author and year of publication	Textbook outcomes	Quality appraisal	
		ISPOR (Yes/Partially/No/NA)	ROBINS-E (Domain 7†)
Endo 2023 [86]	TO^1^: (OR): Low vs. High: 1.37^a^ [1.10–1.63]**	8/1/12/6	High Risk
Munir 2023 [90]	TO^1^: (OR): Low vs. Moderate: 1.55^a^ [1.25–1.92]**; High: 1.67^a^ [1.24–2.25]**	9/1/11/6	Low Risk

*TO* Textbook outcome

^a^ OR [95% CI]

*P value < 0.05, **P value < 0.01, ***P value < 0.001

^1^TO was defined as no 90-day mortality, no unplanned readmission within 30 days after discharge, no prolonged length of stay (LOS), no conversion to open, and R0 resection

^2^TO was defined as R0 resection, adequate lymph node assessment, absence of prolonged length of stay postoperative length of stay (LOS), no unplanned readmission, no 90-day mortality, and initiate guideline compliant adjuvant chemotherap

†Domain 7 = Risk of bias in selection of reported result; Reference categories were underscored if available

#### Mortality outcomes

##### Results at hospital-volume level

In the subgroup of anatomical resections, one study reported statistically significant lower *90-day mortality* in HVHs [67]. Similarly, hospital mortality was lower in HVHs in a 2020 study [2] and in an incremental analysis [74]. However, no significant association was found in a study that defined HVHs as >13 resections per year [73] (Table 3).

**Table 3:** Mortality outcomes (hospital-volume level, multi-page table)

First author and year of publication	Mortality outcomes	Quality appraisal	
		ISPOR (Yes/Partially/No/NA)	ROBINS-E (Domain 7†)
Anatomical resections			
Hunger 2019 [73]	Hospital mortality: (OR): 1–2 vs. 3–5: 0.94^a^ [0.61–1.44]; 6–12: 1.19 ^a^ [0.78–1.84]; 13–40: 1.25 ^a^ [0.82–1.94]	10/8/3/6	High Risk
Krautz 2020 [2]	Hospital mortality: Major hepatectomies: (OR): Very low vs. Low: -; Medium: 0.73^a^ [0.6–0.9]; High: 0.65^a^ [0.5–0.8]; Very high: 0.59^a^ [0.4–0.9]| FTR: (%): Very low: 29.38^d^ [26.7–32.2]; Low : 27.13^d^ [23.9–30.7]; Medium: 28.05^d^ [24.8–31.6]; 41–100: 24.0^d^ [21.7–26.5]; High: 21.38^d^ [19.2–23.8]| Hospital mortality: Minor hepatectomies (OR): Very low vs. Low: -; Medium: 0.73^a^ [0.6–0.9]; High: 0.65^a^ [0.5–0.8]; Very high: 0.59^a^ [0.4–0.9]| FTR: (%): Very low: 17.9^d^ [15.6–20.5]; Low: 15.33^d^ [12.0–19.3]; Medium: 14.0^d^ [10.6–18.1]; High: 17.1^d^ [13.7–21.1]; Very high: 13.54^d^ [10.1–17.7]	11/3/7/6	High Risk
Kohn 2010 [74]	Hospital mortality: (OR): Incremental effect: 0.975^a^ [0.967–0.983]***	9/1/11/6	High Risk
Diggs 2021 [67]	Mortality: (90 days): RR: High vs. Low: 1.60^c^ [1.25–2.05]***	8/2/11/6	High Risk
Anatomical and non-anatomical resections separately			
Hoerger 2023 [89]	Mortality: (OR): Major hepatectomy: (90 day): Low vs. High: 0.62^a^ [0.49–0.80]***; (30 day): Low vs. High: 0.58^a^ [0.41–0.75]***| Mortality: (OR): Any hepatectomy: (90 days): Low vs. High: 0.67^a^ [0.52–0.87]**; (30 days): Low vs. High: 0.55^a^ [0.42–0.73]***	11/1/9/6	Low Risk
Eguia 2021 [79]	Hospital mortality: (OR): Lobectomy: Low-to-high *p*<0.05 vs. Very high: 0.47^a^ [0.12 −1.82]| Partial hepatectomy: Low-to-high *p*<0.05 vs. Very high: 0.66^a^ [0.16–2.67]	11/1/9/6	High Risk
Magnin 2023 [62]	FTR: (OR): Low vs. High: 0.79^a^ [0.65–0.97]***| Hospital mortality: (OR): Low vs. High: 0.74^a^ [0.58–0.93]*** FTR: (OR): Minor hepatectomy: Low vs. High: 0.82^a^ [0.67–0.99]***; Hospital mortality: Low vs. High: 0.80^a^ [0.65–0.99]*| FTR: (OR): Major hepatectomy: Low vs. High: 0.91^a^ [0.70–1.19]; Hospital mortality: Low vs. High: 0.83^a^ [0.63–1.10]	8/3/10/6	High Risk
Yasunaga 2012 [58]	All-cause mortality: (OR): (30-day): Very low vs. Low: 0.70^a^ [0.48–1.02]; High: 0.52^a^ [0.34–0.81], *p*<0.01; Very high: 0.16^a^ [0.09–0.30], *p*<0.001	8/1/12/6	High Risk
Anatomical and non-anatomical resections combined			
Ardito 2019 [65]	Mortality: (OR): (90-day): High vs. Low: 5.625^a^ [1.050–30.131]*; Intermediate: 7.119^a^ [3.047–16-632]**/FTR: High vs. Low-intermediate: 5.095^a^ [1.878–13.821]**| FTR: High vs. Low-intermediate: 5.995^a^ [1.961–18.328]**; (After PSM): High vs. Low-intermediate: 5.069^a^ [1.409–18.232]*	9/4/8/6	Low Risk
Buettner 2016 [80]	Postoperative mortality: (OR): High vs. Low: 2.13^a^ [1.31–3.47]**; Intermediate: 2.00^a^ [1.24–3.21]**| FTR: High vs. Low: 2.15^a^ [1.33–3.48]**; Intermediate: 2.04^a^ [1.25–3.33]**	12/2/7/6	High Risk
Gani 2016 [55]	Postoperative mortality: (%): Low: 9.0^d^; Intermediate: 7.6^d^; High: 1.3^d^ *| FTR: Low: 16.6^d^; Intermediate: 24.7^d^; High: 15.1^d^ **	11/4/6/6	High Risk
Shaw 2013 [71]	Hospital Mortality: (OR): Low vs. Intermediate -; High: 0.44^a^ [0.13–0.56]	11/4/6/6	High Risk
El Amrani 2019 [69]	Mortality: (OR): (90-day): High vs. Low: 1.34^a^ [1.07–1.67]**	10/2/9/6	Low Risk
Gani 2017 [54]	Postoperative mortality: (OR): Low vs. Intermediate: 0.78^a^ [0.61–0.99]*; High: 0.68^a^ [0.51–0.92]**	11/2/8/6	High Risk
Okinaga 2018 [56]	Mortality: (OR): (90-day): Very low vs. Low: 0.84^a^ [0.67 - 1.05]; High: 0.60^a^ [0.47 - 0.78]***; Very high: 0.36^a^ [0.27 - 0.49]***	9/1/11/6	Low Risk
Beal 2019 [78]	OS: (median): Q1 (lowest): 30.4; Q2: median 31.84; Q3: median 37.65; Q4: median 51.7*| HR: (30 to 90 day mortality): Q1 (lowest) vs. Q2: 1.11^b^ [0.90–1.37]; Q3: 0.97^b^ [0.79–1.18]; Q4: 0.69^b^ [0.58–0.82]**| ***Per 10 cases***: 0.981^b^ [0.977–0.984]**	11/0/10/6	High Risk
Farges 2012 [88]	Mortality: (OR): (90-day): ≤ 5 vs.6–10: 0.81^a^ [0.56–1.16]; 11–25: 0.50^a^ [0.35–0.72]***; 26–50: 0.58^a^ [0.39–0.86]**; 51–100: 0.51^a^ [0.34–0.78]**; >100: 0.53^a^ [0.34–0.82]** Hospital mortality: ≤ 5 vs. 6–10: 0.89^a^ [0.53–1.49]; 11–25: 0.56^a^ [0.34–0.93]*; 26–50: 0.54^a^ [0.30–0.96]*; 51–100: 0.52^a^ [0.29–0.94]*; >100: 0.56^a^ [0.30–0.95]*	10/1/10/6	High Risk
Siegel 2021 [68]	OS: (HR): High vs. Low: 0.74^b^ [0.64–0.87.64.87]***| (excluding 30-day): High vs. Low: 0.76^b^ [0.65–0.89]***| (excluding 90-day): High vs. Low: 0.75^b^ [0.64–0.88]***	9/3/9/6	High Risk
Chiu 2015 [52]	Mortality: (HR): (90-day): Low vs. High: 0.79^b^ [0.698–0.887]***| (5-years): Low vs. High: 0.91^b^ [0.873–0.970]**| Hospital mortality^1^: (%): Low: 3.4^d^; High: 1.4^d^ ***| 5-year mortality^1^: Low: 41.7^d^;High: 32.8^d^ ***| OS^1^: (months): Low: 68.6^e^ (0.6); High: 77.1^e^ (0.7)***	9/2/10/6	High Risk
Lee 2019 [87]	Mortality: (OR): (30-day): Low vs. High: 0.68^a^ [0.40–1.16]| (90-day): Low vs. High: 0.68^a^ [0.46–1.01]| (1-year): Low vs. High: 0.95^a^ [0.79–1.14]	7/4/10/6	High Risk
Chang 2014 [63]	Mortality: (OR): (30-day): High vs. Low: 1.50^b^ [1.09–2.07]**| (3-months): High vs. Low: 1.56^b^ [1.30–1.86]***| (1-year): High vs. Low: 1.33^b^ [1.21–1.46]**	8/3/10/6	High Risk
Endo 2023^2^ [86]	OS: (HR): Low vs. High: 0.83^b^ [0.69–0.99]*	8/1/12/6	High Risk
Sato 2012 [57]	Hospital mortality: (OR): High vs. Low: 2.74^a^ [1.74–4.30]***; Intermediate: 1.45^a^ [0.88–2.38]	8/1/12/6	High Risk
Spolverato 2014 [83]	Hospital mortality: (OR): High vs. Low: 1.50^a^ [1.13–1.99]**; Intermediate: 1.73^a^ [1.25–2.39]**| FTR: High vs. Low: 1.40^a^ [1.02–1.93]**; Intermediate: -	8/1/12/6	High Risk
Idrees 2018 [51]	FTR: (OR): Low vs. Medium: 0.95^a^ [0.61–1.48]; High: 0.69^a^ [0.37–1.27]	7/3/11/6	High Risk
Chapman 2017^3^ [82]	Survival: High vs. Low CCCP: 1.14^b^ [1.05–1.22]***| High vs. Low ACP: 1.13^b^ [1.06–1.20]***	6/3/12/6	High Risk

*FTR* Failure to rescue, *OS* Overall survival, *ACP* Academic cancer programs, *CCCP* Comprehensive community cancer program

^a^ OR [95% CI]

^b^ HR [95% CI]

^c^ RR [95% CI]

^d^ Rate in % [95% CI]

^e^ mean (SD)

*P value < 0.05, **P value < 0.01, ***P value < 0.001

^1^ Outcomes from Lu et al. 2014

^2^ Minimal invasive cases/6 years

^3^ 1-year, 5-year, 10-year survival in digital appendix

† Domain 7 = Risk of bias in selection of reported result; Reference categories were underscored if available

Among studies reporting separate results for anatomical and non-anatomical resections, three out of four studies reported statistically significant lower hospital, 30-day, and 90-day mortality, and overall survival (OS) in HVHs. A 2012 Japanese study found lower 30-day mortality in HVHs (≥36 cases/year) [58]. A 2023 US study found significantly lower 30-day and 90-day mortality after lobectomy in HVHs (≥46 cases/year). 

Several studies reported aggregated results for both anatomical and non-anatomical resections. Two examined 30-day mortality after hepatectomies [63, 87]. One study found higher mortality in LVHs (< 245 cases/year) [63], while a more recent study found no significant association favoring HVHs (≥11 cases/year) [87]. Multiple studies assessed 90-day mortality [52, 56, 63, 65, 69, 78, 87, 88]. All but one US study from 2019 showed a significant inverse relationship between hospital-volume and mortality [87], and one study demonstrated this effect across continuous volumes [78]. Two studies analyzed intermediate and long-term mortality, comparing LVHs (<245, <100 procedures/year) and HVHs (≥245, ≥100 procedures/year) [52, 63]. One reported a 50% higher hazard of 1-year mortality in LVHs [63], while the other reported significantly lower adjusted mortality rates in HVH [52]. 

Five studies reported improved OS with higher volumes [52, 68, 86, 82, 78]. A 2017 study associated LVHs with decreased 1-, 5-, and 10-year OS [82], while a 2015 Taiwanese study reported a statistically significant OS benefit in HVHs (≥100 hepatectomies/year) [52]. A 2019 study showed improved survival with incremental volume analysis [78], and a 2023 study reported higher OS in hospitals performing ≥50 MIH over 6 years [86]. In contrast, a 2019 study found that ≥11 annual cases were not significantly associated with higher 1-year OS [87], and a 2021 study reported higher OS in LVHs (<466 cases/10 years) [68].

The results are stratified by mortality type in Appendix 5.

##### Results at surgeon-volume level

Three studies assessed *short-, intermediate, and long-term mortalit*y in relation to surgeon-volume [63, 52, 77], combining anatomical and non-anatomical resections in their analyses (Table 4). LVS (<25–29 cases/year) were significantly associated with higher 30- and 90-day mortality, while HVSs (≥25–30 cases/year) showed significantly lower 90-day and long-term mortality [52, 63], supported by a propensity score matched (PSM) analysis in the latter study [52]. Additionally, a 2021 study focusing exclusively on MILS procedures reported significantly improved 30-day and 90-day mortality for HVSs (≥8 cases/year) [77], and two others also demonstrated lower hospital mortality and FTR rates for HVSs (≥16 [80], >38 [71] cases/year). The results are stratified by mortality type in Appendix 6. 

**Table 4:** Mortality outcomes (surgeon-volume level)

First author and year of publication	Mortality outcomes	Quality appraisal	
		ISPOR (Yes/Partially/No/NA)	ROBINS-E (Domain 7†)
Anatomical and non-anatomical resections combined			
Buettner 2016 [80]	Postoperative mortality: (OS): High vs. Low: 3.01^a^ [1.80–5.04]***; Intermediate: 2.56^a^ [1.54–4.26]***| FTR: High vs. Low: 3.42^a^ [1.98–5.93]***; Intermediate: 3.08^a^ [1.77–5.34]***	12/2/7/6	High Risk
Shaw 2013 [71]	Hospital mortality: (OR): Low vs. Intermediate: -; High: 0.55^a^ [0.33–0.89]	11/4/6/6	High Risk
Chiu 2015 [52]	Mortality: (HR): (90-day): Low vs. High: 0.86^b^ [0.822–0.902]***| (5-year): Low vs. High: 0.84^b^ [0.799–0.878]***| Hospital mortality^1,2^: (%): Low: 4.2^d^; High: 1.0^d^***| (5-year)^1,2^: Low: 43.9^d^; High: 30.2^d^| OS^1,2^: (months): Low: 66.9^e^ (0.7); High: 78.5^e^ (0.7)**	9/2/10/6	High Risk
Chang 2014 [63]	Mortality: (OS): (30-day): High vs. Low: 1.64^b^ [1.12–2.41]*| (3-month): High vs. Low: 1.62^b^ [1.31–2.00]**| (1-year): High vs. Low: 1.33^b^ [1.21–1.46]**	8/3/10/6	High Risk
Tsilimigras 2021 [77]	Mortality: (OR): (30-day): Average (minimally invasive only) vs. Above average: -; High: 0.59^a^ [0.45–0.78]| (90-day): Average (minimally invasive only) vs. Above average: -; High: 0.64^a^ [0.51–0.79]	8/3/10/6	High Risk

*FTR *Failure to rescue, *OS* Overall survival

^a^ OR [95% CI]

^b^ HR [95% CI]

^c^ RR [95% CI]

^d^ Rate in % [95% CI]

^e^ mean (SD)

^1^ Outcomes from Lu et al. 2014

^2^ Propensity-score matched cohort

*P value < 0.05, **P value < 0.01, ***P value < 0.001

† Domain 7 = Risk of bias in selection of reported result; Reference categories were underscored if available

#### Morbidity outcomes

##### Results at hospital-volume level

Three studies focused exclusively on anatomical liver resections [72, 74, 75]. One study reported an inverse association between continuous hospital-volume and the occurrence of any complication, although the functional form of this relationship was not specified (Table 5) [74]. Two US studies assessed specific complications following lobectomies: one found no association with high-transfusion use [72], while the other reported lower 30-day readmission in HVHs (30–86 cases/year) [75].

**Table 5:** Morbidity outcomes (hospital-volume level, multi-page table

Hospital-volume				
First author and year of publication	Outcome definition	Results	Quality appraisal	
			ISPOR (Yes/Partially/No/NA)	ROBINS-E (Domain 7†)
Anatomical resections				
Sutton 2016 [75]	30-day readmission	Low vs. Medium: (OR): 0.857^a^ [0.683–1.076]; High: 0.672^a^ [0.532–0.849]***	10/2/9/6	High Risk
Dhar 2019 [72]	High transfusion use^1^	(OR): High vs. Low: 1.30^a^ [1.01–1.68]*; Medium: 0.86^a^ [0.68–1.09]|(OR): High vs. Low: 1.11^a^ [0.88–1.42]; Medium: 0.87^a^ [0.69–1.10]	9/1/11/6	High Risk
Kohn 2010 [74]	Any complication^2^	Incremental effect: (OR): 0.992^a^ [0.987–0.996]**	9/1/11/6	High Risk
Anatomical and non-anatomical liver resections separately				
Hoerger 2023 [89]	30-day readmission/positive surgical margins	All hepatectomies: (OR): 30-day readmission:< 75^th^ vs. > 75^th^: 1.08^a^ [0.71–1.63]| Positive surgical margins:< 75^th^ vs. > 75^th^: 0.79^a^ [0.56–1.12]| Lobectomy: (OR): 30-day readmission:< 75^th^ vs. > 75^th^: 0.92^a^ [0.62–1.39]| Positive surgical margins:< 75^th^ vs. > 75^th^: 0.91^a^ [0.66–1.25]	11/1/9/6	Low Risk
Eguia 2021 [79]	Postoperative complications^2^	Laparoscopic lobectomy: (OR): Low-to-High vs. Very-high: 0.72^a^ [0.40 1.32] Laparoscopic partial hepatectomy: (OR): Low-to-high vs. Very-high: 0.48^a^ [0.33–0.69]***	11/1/9/6	High Risk
Anatomical and non-anatomical liver resections combined				
Ardito 2020 [65]	Major complications3	(OR): High vs. Low: 2.622^a^ [1.278–5.378]** Intermediate: 3.121^a^ [2.073–4.699]***| Low-intermediate vs. High: 2.981^a,4^ [1.758–5.055]**	9/4/8/6	Low Risk
Gani 2016 [55]	Postoperative complications^2^	(%): Low: 26.0^d^; Intermediate: 25.0^d^; High: 20.5^d^***	11/4/6/6	High Risk
Gani 2017 [54]	Postoperative complications^2^	(OR): Low vs. Intermediate: 0.83^a^ [0.74–0.94]**; High: 0.71^a^ [0.59–0.86]***	11/2/8/6	High Risk
Okinaga 2018 [56]	Postoperative morbidity^2^	(OR): Very low vs. Low: 1.00^a^ [0.85–1.17]; High: 1.07^a^ [0.81–1.45]; Very high: 1.02^a^ [0.69–1.52]	9/1/11/6	Low Risk
Beal 2019 [78]	Positive surgical margin	(OR): First (lowest) vs. Second: 1.18^a^ [0.70–1.98]; Third: 0.97^a^ [0.60–1.58]; Fourth: 0.72^a^ [0.47–1.112]| Per 10 cases: 0.976^a^ [0.967–0.985]***	11/0/10/6	High Risk
Shaw 2013 [71]	Morbidity^2^/ 30-day readmission	(OR): Morbidity: Low vs. Intermediate: -; High: 0.53^a^ [0.38–0.71]| 30-day readmission: Low vs. Intermediate: -; High: 0.73^a^ [0.64–0.91]	8/1/12/6	High Risk
Spolverato 2014 [83]	Any complication^2^	High vs. Low: 1. 1.17 [1.03, 1.33]*; Intermediate: 1.21 [1.05, 1.39]*	8/1/12/6	High Risk
Lee 2019 [87]	30-day readmission/positive surgical margins	(OR): 30-day readmission: Low vs. High:1.39^a^ [0.73–2.65]| Positive surgical margins: Low vs. High: 0.87^a^ [0.64–1.19]	7/4/10/6	High Risk

^a^ OR [95% CI]

^b^ HR [95% CI]

^c^ RR [95% CI]

^d^ Rate in % [95% CI]

^1^ >5 units

^2^ List of specific complications in digital appendix

^3^ Clavien Dindo ³ 3

^4^ Propensity-score matched

*P value < 0.05, **P value < 0.01, ***P value < 0.001

† Domain 7 = Risk of bias in selection of reported result; Reference categories were underscored if available

Two studies stratified outcomes by anatomical versus non-anatomical resections [79, 89]. One found no significant relationship between HVHs and various postoperative outcomes [89]. The second study reported fewer complications in partial hepatectomies performed in very-HVHs (>314 annual procedures), but not in lobectomies [79].

Several studies examined morbidity outcomes across combined anatomical and non-anatomical resections [54–56, 65, 71, 78, 83, 87], with many showing significantly lower complication rates in HVHs. A 2020 study found a nearly threefold increase in major complications in LVHs (≤ 49 cases/year), confirmed in a PSM analysis [65]. Similar findings of reduced complications in HVHs (≥ 15, >43, ≥ 45, >100 cases/year) were reported in studies published in 2013, 2014, 2016 [55, 71, 83 ]. 

In contrast, a 2018 Japanese study found no statistically significant associations between HVHs (>52 cases/year) and postoperative complications or morbidity [56]. Two studies specifically examined 30-day readmission rates, with mixed results depending on volume thresholds [71].

The association between hospital-volume and positive surgical margins was investigated in three studies [89, 87 ,78]. No significant relationships were found, except in one study that reported significantly reduced odds of positive surgical margins for every additional 10 cases per year [78]. 

##### Results at surgeon-volume level

Three studies analyzed morbidity-related outcomes related to surgeon-volume [61, 71, 72]. Among these, two were based on University HealthSystem Consortium (UHC) data, with one study focusing exclusively on anatomical liver resections [71, 72] (Table 6). In the 2019 study, no significant association was found between LVSs (1–4 cases/year) and transfusion rates after lobectomy, while multivariable results were reported as non-significant [72]. A 2013 Canadian study found that, although unadjusted analyses favored LVSs, adjusted models indicated a higher complication risk among HVSs [61]. In contrast, another 2013 study reported a nearly twofold lower complication risk for patients treated by HVSs (>38 cases/year) [71]. 

**Table 6:** Morbidity outcomes (surgeon-volume level)

Surgeon-volume				
First author and year of publication	Outcome definition	Results	Quality appraisal	
			ISPOR (Yes/Partially/No/NA)	ROBINS-E (Domain 7†)
Anatomical surgery types				
Dhar 2019 [72]	High transfusion use^1^	(OR): High vs. Low: 0.87^a^ [0.66–1.13]; High: 0.95^a^ [0.74–1.12]	9/1/11/6	High Risk
Anatomical and non-anatomical liver resections combined				
Shaw 2013 [71]	Morbidity^2^/30-day readmission	(OR): Morbidity: Low vs. Intermediate: -; High: 0.56^a^ [0.46–0.80]| 30-day readmission: Low vs. Intermediate: -; High:0.69^a^ [0–56-0.86]	11/4/6/6	High Risk
McColl 2013 [61]	Postoperative complications^2^	(OR): Low vs. High: 1.91^a^ [1.16–3.14]	7/4/10/6	High Risk

^a^ OR [95% CI]

^1^ >5 units

^2^ list of specific complications in digital appendix

† Domain 7 = Risk of bias in selection of reported result; Reference categories were underscored if available

#### Length of stay

##### Results at hospital-volume level

Five studies reported adjusted results on LOS in relation to hospital-volume [52, 55, 89, 87, 79] (Table 7). Two of these studies provided stratified results for anatomical and non-anatomical resections, though findings were inconsistent [89, 79]. One study found no association between LVHs (< 46 cases/year) and prolonged LOS [89], while another reported significantly shorter LOS for partial hepatectomy patients in very HVHs (>314 laparoscopic cases/year) [79].

**Table 7:** Length of stay (hospital-volume level)

Hospital-volume				
First author and year of publication	Outcome definition	Results	Quality appraisal	
			ISPOR (Yes/Partially/No/NA)	ROBINS-E (Domain 7†)
Anatomicaland non-anatomical surgery types separately				
Hoerger 2023 [89]	Prolonged LOS	All hepatectomies: < 75^th^ vs. ≥ 75^th^: 0.99^d^ [0.72–1.37]| Lobectomy: < 75^th^ vs. ≥ 75^th^: 0.91^a^[0.65–1.28]	11/1/9/6	Low Risk
Eguia 2021 [79]	LOS/Prolonged LOS	(log-OR): Laparoscopic lobectomy: LOS: Low-to-high vs. Very-high: −2.26^b^[-5.42-–0.89]| Prolonged LOS: Low-to-high vs. Very-high: 0.73^a^ [0.29–1.82]| Laparoscopic partial hepatectomy: LOS: Low-to-high vs. Very-high: −2.17^b^ [-3.92−-0.42]***| Prolonged LOS: Low-to-high vs. Very-high: 0.57^a^[0.28–1.17]	11/1/9/6	High Risk
Anatomical and non-anatomical liver resections combined				
Gani 2016 [55]	LOS^c^	LOS: Low: 6.2^1,d^; Intermediate: 6.1; High: 7.4| Complication and survived discharge: Low: 11.6^2,d^ Intermediate: 9.9; High: 7.7***| Complications and died during hospital stay: Low: 15.1^3,d^; Intermediate: 13.7; High: 4.9***	11/4/6/6	High Risk
Chiu 2015 [52]	LOS	(Regression weight): Low vs. High: −2.91^c^; SE 0.28***	9/2/10/6	High Risk
Lee 2019 [87]	Length of stay ≥ 7 days	(OR): Low vs. High: 1.06^a^ [0.76–1.47]	7/4/10/6	High Risk

*LOS* Length of stay, *SE* Standard error

^a^ OR [95% CI]

^b^ log OR [95% CI]

^c^ Hierarchical linear regression coefficient

^d^ days *P value < 0.05, **P value < 0.01, ***P value < 0.001

^1^ All included patients

^2^ Patients who developed a complication and survived to discharge

^3^ Patients who developed a complication and died during hospital stay

Note: † Domain 7 = Risk of bias in selection of reported result; Reference categories were underscored if available

Three studies combined anatomical and non-anatomical resections in their analyses [52, 55, 87]. One found significantly shorter LOS in HVHs (≥100 hepatectomies/year) [52], while another observed no overall difference, but among patients with complications who survived to discharge, LOS was shortest in HVHs (≥ 15 cases/year) [55]. A third study found no significant association between extended LOS (≥ 7 days) and HVHs (≥11 cases/year) [87].

##### Results at surgeon-volume level

 LOS was examined in one Taiwanese study from 2015 [52], including partial hepatectomies and lobectomies. Hepatectomies performed by HVSs (≥ 30 hepatectomies/year) were associated with a significantly reduced LOS, with a mean reduction of 2.44 days compared to LVSs (Table 8).

**Table 8:** Length of stay (surgeon-volume level)

Surgeon-volume				
First author and year of publication	Outcome definition	Results	Quality appraisal	
			**ISPOR** (Yes/Partially/No/NA)	**ROBINS-E** (Domain 7†)
Anatomical and non-anatomical liver resections combined				
Chiu 2015 [52]	LOS	(Regression weight): Low vs. High: −2.44^a^; SE 0.26*	9/2/10/6	High Risk

*SE* Standard error

*P value < 0.001

^a^ Hierarchical linear regression coefficient

† Domain 7 = Risk of bias in selection of reported result; Reference categories were underscored if available

#### Summary of findings

An overview of the findings is presented in Table 9, sorted by assessed study quality. The average quality score across all included studies was 10.5 points, which is marked in the table by a bold horizontal line to distinguish studies above and below the average threshold. The scores ranged from 7.5 on the low end to a total of 14 points on the high end, achieved by one study. The summation of ISPOR and ROBINS-E allowed for a maximum of 23 points (omitting NAs). In total, study quality was low to medium, such that higher quality studies above the threshold still normatively belong to a medium study quality. Outcomes were interpreted according to their desirability from the patient’s perspective. For instance, reductions in complications, mortality, or LOS were considered favorable, while increases in TO or readmission were considered favorable.

**Table 9:**
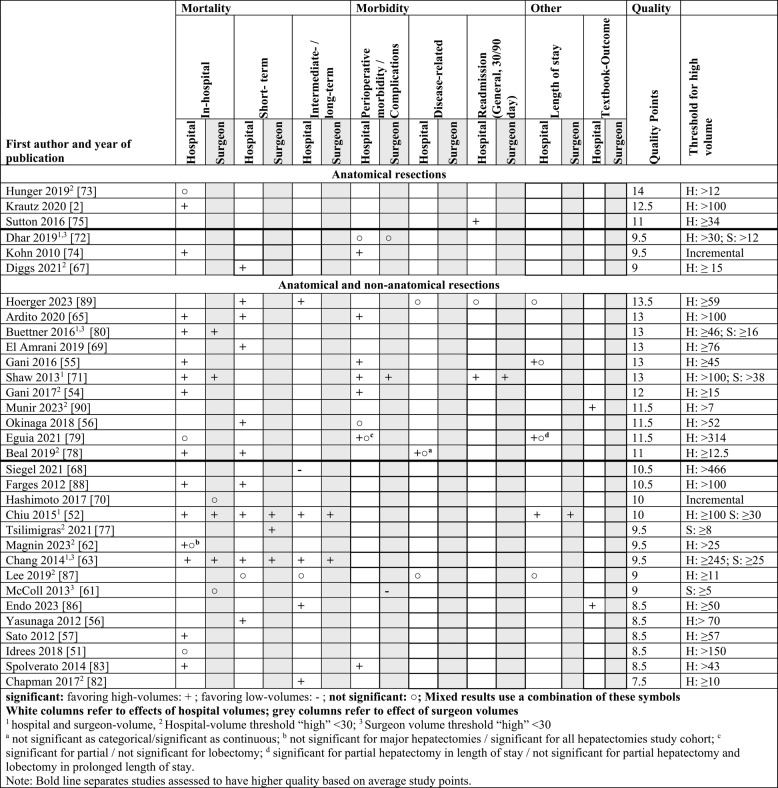
Results overview

To standardize the interpretation of volume-outcome associations across studies, a symbolic coding system was applied to denote the direction and statistical significance of findings at both hospital and surgeon levels. A plus sign (+) indicates a statistically significant association favoring higher volumes, a minus sign (-) represents a significant association favoring lower volumes. For non-significant results, a circle (**○**) was used. Each outcome is stratified by hospital or surgeon-volumes. Symbols in the grey columns refer to surgeon-volume effects, whereas symbols in the white columns represent hospital-volume effects. This coding was applied to facilitate consistent comparison of study outcomes and to highlight exceptions to the commonly observed volume–outcome relationship. The definition of high-volume categories (or continuous volume analyses) are reported for each study. For an additional visual representation, a harvest plot is provided in Appendix 7. Appendix 8 depicts a harvest plot using an alternative specification of the quality score as a sensitivity analysis.

The most frequently reported outcomes were mortality and morbidity. LOS was examined less frequently and mostly in relation to hospital-volume level; only one study considered surgeon-volume. TOs were investigated in two studies focusing on annual hospital-volume. Six studies exclusively investigated anatomical liver resections and reported improved outcomes for mortality and morbidity in favor of HVHs. Most studies included both anatomical and non-anatomical resection and consistently showed that higher volumes at hospital and surgeon level were associated with improved outcomes. Notably, however, two studies reported results suggesting an opposite volume-outcome relationship for morbidity and mortality, indicating worse outcomes in higher-volume hospitals [61, 68]. Among adjusted studies, the thresholds defining HVH/HVS ranged from seven to 466 procedures for hospitals and from five to 38 procedures for surgeons. Studies reporting a significant hospital volume-outcome relationship with hospital mortality included thresholds between 12.5 and 100 cases, while those reporting no association used thresholds between 12 and 314. For short-term mortality, studies demonstrating a significant association defined HVHs with thresholds between 12.5 and 245 procedures. Only one study, using a threshold of 11, found no association. Notably, this study also had the lowest threshold for intermediate-/long-term mortality and likewise reported no effect. For this outcome, the study applying the highest threshold among all included studies identified a negative volume-outcome relationship. Regarding perioperative complications and morbidity, two studies reported no association, defining HVHs as >30 and>52 procedures, respectively, while one study using a high threshold of 314 procedures reported mixed results, as certain complications showed no association. Excluding incremental analyses, overall median HVH threshold was 48 procedures (mean of 78.6). Among higher quality studies, the median HVH threshold was 49 (mean of 69.5), while the remaining studies had a median threshold of 46.5 (mean of 86.6).

## Discussion

In this review, we found evidence supporting the volume-outcome relationships for some outcomes following liver resections, both at hospital and surgeon level. However, the associations remain heterogeneous, particularly with respect to study quality and resection types analyzed. Across studies that included both anatomical and non-anatomical resections, the volume-outcome association was most consistent for in-hospital and short-term mortality, particularly at hospital level.

Most studies focused on hospital-level volume; results at surgeon level were often nested in studies analyzing both hospital and surgeon level. Only a few studies examined outcomes following anatomical resections exclusively, limiting the generalizability of findings for this subgroup. Among these, one higher quality study did not find a significant association between hospital-volume and mortality [73], whereas another study of comparable quality contradicted these findings and applied a substantially higher volume threshold [2]. The results and synthesis on short-term mortality confirm the findings of previous studies, reporting a significant pooled OR of 1.5 (95%-CI: 1.0–2.1), p=0.03 in favor of high volume based on adjusted analyses [33]. A recent Bayesian meta-analysis showed increased overall short-term mortality in LVH (OR: 0.52; 95%-CI: 0.41–0.65) [37]. Interestingly, this analysis found no significant differences between hospital volumes in a subgroup of studies that defined HVH as those performing ≥ 50 resections per year.

Intermediate and long-term mortality were assessed in a limited number of studies. While most found a significant association with volume, these studies tended to exhibit lower study quality. One higher-quality study reported a significant volume-mortality relationship at hospital level [89]. However, the next highest quality study contradicted these results, reporting worse outcomes in HVHs [68], suggesting a reverse volume–outcome effect. The same study also reported no significant association between hospital volume and morbidity outcomes as well as LOS [89]. 

Some studies of good or acceptable quality confirmed a volume-outcome association for perioperative morbidity or complications [54, 55, 65, 71], while others yielded mixed results, often deploying different volume definitions or subgroup analyses [55, 78, 79]. However, these findings contrast with a 2024 Bayesian meta-analysis including six studies on postoperative complications, which reported overall lower morbidity rates in LVH, although the results were not statistically significant. For LOS and readmission, the overall evidence was inconclusive in contrast to the Bayesian meta-analysis, which found significantly reduced LOS in nine included studies [37]. Notably, this analysis included studies analyzing LOS published before 2010, although many overlapped with those included in this review. Nevertheless, most of said the studies in the meta-analysis presented unadjusted results, which may explain this discrepancy.

Two higher-quality studies provided conflicting findings on readmissions [75, 89], but only few studies assessed this outcome. LOS was more commonly reported but generally showed no consistent association with volume. This aligns with an earlier review, which found favorable volume effects in only two out of eight included studies, one of which reported limited clinical relevance with a median reduction of just one-day [33]. While the more recent meta-analysis also reported significantly reduced LOS in HVH, the magnitude of reduction (1.24 days) was comparable to that observed in the earlier analysis. Regarding TO, two studies of higher or acceptable quality reported improvement in favor of HVHs [90, 86]. 

Overall, the evidence base was skewed towards hospital volume in terms of both quantity and quality of studies. The relative scarcity of studies at surgeon level may be explained by a more prevailing theory for the analysis of hospital volumes and data availability. Hospital-level volume data are more accessible in retrospective databases and registries. The small number of studies focusing solely on anatomical resections also limits conclusions for this subgroup. Some discrepancies were identified in comparison to the most recent meta-analysis available to date, particularly regarding morbidity outcomes. While the discussed meta-analyses account for certain sources of heterogeneity, this review is the first to stratify liver resection by anatomical and non-anatomical resections, thereby highlighting evidence gaps related to surgical complexity and extent. In this rapid review, studies published before 2010 were excluded to reflect relevant policy changes and advancements in surgical technique. During this period, HCC incidence and mortality began to plateau and decline [45]. Accordingly, this review provides an up-to-date synthesis of the available evidence but also raises the question of whether observed differences from previous meta-analyses and reviews result from time-dependent multifactorial trends and how long-term volume effects can be distinguished from general improvements in surgical care. Furthermore, some of the observed discrepancies may be attributable to the absence of additional stratification by study quality. Yet, the present synthesis is highly sensitive to assessed study quality. Applying a threshold based on the mean quality scores, positive evidence emerged for a volume-outcome relationship at hospital volume regarding in-hospital and short-term mortality, as well as perioperative complications. While this tendency is also present at the surgeon level, this exposure remains underexplored. Additionally, evidence for intermediate and long-term mortality remains limited by the overall smaller number and lower quality of available studies. In summary, results for disease-related morbidity, readmissions and LOS did not show a consistent association with volume.

## Limitations

Several included studies relied on the same underlying database, creating a potential risk of overlapping patient populations, which may introduce correlated evidence. Nevertheless, studies using the Nationwide Inpatient Sample (NIS) across partially overlapping periods were retained due to meaningful methodological differences. For example, Buettner et al. 2016 [80] investigated the association between surgeon and hospital procedure volume and postoperative outcomes. In contrast, Gani et al. 2017 [54] and Gani et al. 2016 [55] examined hospital-volume alone, but differed in their volume threshold definitions and primary endpoints, with the former focusing on mortality and morbidity and the latter additionally analyzing length of stay. Idrees et al. 2018 [51] included the largest cohort and applied a substantially higher high-volume threshold compared to the other studies. These distinct volume stratifications, inclusion criteria, and outcome measures justied their separate inclusion in the synthesis.

An important limitation lies in the inconsistent and arbitrary definitions of volume thresholds, which varied widely. Multiple studies deployed relatively low thresholds for analysis [54, 60, 61, 62, 63, 66, 67, 72, 76, 77, 90, 87, 82, 78, 80], which may have led to the omission of effects observable at higher volume levels. The choice of volume categories is central to volume-outcome analyses and may depend on national standards, the volume of surgeries available in the data and hospital distribution at the regional level. Similar variability has been observed in studies of other surgical procedures [23, 61, 87,78]. Analyses using low thresholds may fail to capture effects that emerge beyond certain volume levels, while excessively high cutoffs may overshoot the point of diminishing returns in the association between volume and outcomes, as shown in an analysis of volume-outcomes for bariatric surgery [84]. This phenomenon may also apply here. While higher quality studies tended to use higher thresholds, some of the studies showing no or even negative association between volume and outcomes used the highest thresholds [51, 68, 79]. 

Although this represents an issue of calibration and operationalization of exposure, this analysis observed that most included studies still favored higher volumes across a variety of definitions. Higher quality studies were also more likely to use well-defined and transparently reported volume thresholds, which positively influenced their quality assessment under ROBINS-E criteria. For example, a 2020 study excluded LVHs performing less than 20 hepatectomies per year based on previously published minimum-volume standards for liver surgery hospitals in Italy [65]. Other studies applied statistical methods to determine cutoffs, as observed in a 2019 French study and a 2022 US study [69, 89]. While these methods offer a data-driven approach to identifying volume thresholds, they may introduce bias and limit external validity when applied to the same dataset used for effect estimation. Volume definitions may also explain the unexpected findings in studies reporting better outcomes in LVHs or by LVSs. However, these results are more likely to be attributed to factors such as self-selection of patients or referral bias, that could be addressed using quasi-experimental designs such as difference-in-difference, stepped-wedge or target emulation trials around national or regional minimum-volume policies. Overall, only five studies with adjusted results were assessed as having a low risk of reporting bias. ISPOR criteria frequently associated with lower study quality included missing description of data reliability, lack of a predefined analysis plan, inadequate handling of censoring, and insufficient reporting of statistical methods. 

Despite the high impact of liver surgery for patients, none of the studies examined HRQoL as an outcome. Measuring HRQoL in retrospective observational studies is challenging due to the reliance on existing data sources such as cancer registries or inpatient records [92]. This represents a substantial empirical gap in literature, which may be addressed with the implementation of pragmatic HRQoL measurement strategies in registries, for example in the context of aftercare [93] or by purposely designed primary studies. Even for more frequently analyzed outcomes, such as perioperative morbidity, heterogeneity persists in outcome definitions and perioperative care protocols across centers and countries, which may introduce variability that is falsely attributed to volume effects.

All studies included in this review were retrospective observational studies, based on routine data or data from registries such as cancer databases. The absence of RCTs is a notable concern, potentially introducing bias [94] and limiting the validity of the results. Although the use of hospital-volume as a proxy for overall experience is common, these studies often lacked adjustment for surgeon experience and volumes on an individual level. Only a few studies in this review adjusted simultaneously for surgeon characteristics and hospital-volume strata, and none modeled a hierarchical structure of surgeon-volume nested within hospital-volume. Even among multivariable-adjusted studies, residual confounding likely remains, as micro-level patient characteristics are often unavailable in registry data. Overall, only two studies supplemented their analyses with causal frameworks using PSM [52, 65]. Hence, most included studies and by extension the results of this review are limited to associative results, with unclear causality. 

Over 50% of the studies relied on US data. The predominance of non-European studies may limit transferability of findings to European healthcare systems, given structural and systemic differences such as provider organization and funding models [95].

Another limitation of this study relates to the inherent methodological limitations of rapid reviews. A single reviewer extracted the data, while another performed the quality assessment. Although the extracted data was checked for accuracy, the risk of single-reviewer bias in screening and quality assessment can occur [85]. Furthermore, rapid reviews have narrower database coverage and constrain depth of synthesis due to heterogeneity and time constraints. The studies often grouped or combined multiple indications, which were too fragmented for subgroup analysis. Consequently, this review is not equipped to recommend an optimal volume threshold for policy implementation. While this constraint could be addressed by deploying meta-analysis, the actual implementation of thresholds for policy may depend on factors beyond a threshold calculated from a summary of the literature, such as economic considerations, available resources, alternative certification processes, political and distributive goals [22, 23]. Policymakers have faced similar challenges in the past, often combining available literature with expert consensus to establish minimum-volume standards [22, 23].

Additional limitations attributable to the review process include decisions on the full text level made by a single reviewer, increasing the potential risk of selection bias, although uncertain cases were discussed with researchers. In some cases, information was not reported in the studies, limiting synthesis. No additional search for grey literature was conducted beyond trial registries. However, reference list screening of systematic reviews yielded additional eligible studies. The limitation to English or German-language studies may have further limited the scope of included evidence. Finally, the quality assessment tools used in this study lacked a summary score. The score calculated in this review used a summation of arbitrary ISPOR points and ROBINS-E domain 7 evaluation.

While this novel pragmatic approach was consistently applied and allowed relative comparison across studies, it relied on arbitrary point allocation. Only five studies were positively affected by the aggregation of ROBINS-I domain 7. A sensitivity analysis omitting this summation produced minimal differences in the results supporting the robustness of the findings.

## Conclusion

A total of 38 relevant studies published between 2010 and 2023 were included in this review. More than half of the studies were based on data from the US, and all studies employed a retrospective study design. The findings from 32 risk-adjusted studies were synthesized. Overall, the evidence indicates improved patient-relevant outcomes with increasing hospital-volume, while considerably fewer studies analyzed surgeon-volume. Quality-stratified evidence and subgroup specific analyses suggest that volume-outcome relationship depends on procedure type, chosen volume thresholds, and the definition of outcome parameters. For instance, various aspects of post- and peri-operative complications showed limited association with annual caseload, whereas evidence was found for the association between higher volume and in-hospital mortality and FTR. Importantly, this review and previous work highlight a persistent evidence gap regarding long-term outcomes, HRQoL, disease-specific morbidity, and exposure on surgeon-volume level. The existing evidence base is largely observational and heterogeneous in design and analytic approach. While these limitations reduce causal interpretability, the overall findings suggest that implementing minimum-volume standards for anatomical and non-anatomical liver resections could improve patient outcomes. Policymakers should consider procedure-specific thresholds that reflect surgical complexity and local hospital capacities. The review thereby contributes to policymaking by informing and supporting expert opinion. Future research should further explore volume-outcome associations specifically for anatomical liver resections, justify or derive their used volume thresholds (including surgeon-level volumes), and place greater emphasis on transparently reported patient-centered outcomes such as HRQoL, which remain underrepresented in the current literature.

## Supplementary Information

Supplementary Material 1.

## Data Availability

The data extracted in this work is available in the publication, appendices and on reasonable request to the corresponding author.

## Abbreviations

PHC: Perihilar Cholangiocarcinoma
PRISMA: Preferred Reporting Items for Systematic Review and Meta-Analyses
PICOS: Population, Intervention, Context, Outcomes, Study Design
DRKS: German Clinical Study Register
ISPOR: International Society for Pharmacoeconomics and Outcome Research
ROBINS-E: Risk of Bias in Non-randomized Studies – of Exposure
PRESS: Peer Review of Electronic Search Strategies
MeSH: Medical subject headings
LOS: Length of stay
FTR: Failure to rescue
US: United states
DPC: Diagnosis Procedure Combination
TO: Textbook outcome
ICU: Intensive care unit
MIH: Minimally invasive hepatectomy
HRQoL: Health-related quality of life
HVH/LVH: High volume hospital / Low volume hospital
HVS/LVS: High volume surgeon / Low volume surgeon
NCDB: National Cancer Database
NHI: National health insurance
OS: Overall survival
UHC: University HealthSystem Consortium
RCT: Randomized controlled trial
PSM: Propensity score matching
