# Correction: Volume–outcome relationship in anatomical and non-anatomical liver resections: a rapid systematic review

**DOI:** 10.1186/s12876-026-04774-w

**Published:** 2026-04-13

**Authors:** Alessandro Campione, Julian Modrow, Helene Eckhardt, Cinara Paul, Ulrike Nimptsch, Cornelia Henschke

**Affiliations:** 1 Department of Health Care Management, Technische Universität Berlin, H80, Straße des 17. Juni 135, Berlin, 10623 Germany; 2 Institute of General Practice and Interprofessional Care, Faculty of Medicine, University Hospital Tübingen, Eberhard Karls Universität Tübingen, Tübingen, Germany

**Correction**: **BMC Gastroenterol 26**,** 178 (2026)**

**https://doi.org/10.1186/s12876-025-04490-x**

Following publication of the original article it was reported that, due to a typesetting error on the part of the publisher, Tables 1, 2, 3, 4, 5, 6, 7, 8 and 9 contained citation and formatting errors.

In the original publication several studies listed had multiple citations associated with them. The erroneous citations have been removed and the formatting errors have been corrected. The correction does not influence the conclusions or results of the original publication.

The original publication with the erroneous tables is included in this Correction as a supplementary file for reference.

The original article has been updated.

## Supplementary Information

Supplementary material 1: Originally published article

