# Supplementary material for: Volume–outcome relationship in anatomical and non-anatomical liver resections: a rapid systematic review
Source: BMC Gastroenterol. 2026 Feb 25;26:178. doi: 10.1186/s12876-025-04490-x (PMC13003663; doi:10.1186/s12876-025-04490-x)
Supplement: Supplementary file 1 — Supplementary Material 1. [file 12876_2025_4490_MOESM1_ESM.docx]

Appendix

Appendix 1: Search strategies for search engines and study registries

| **PubMed:** | |
| --- | --- |
| # 1 | "segmentectom*"[tiab] OR "hemihepat*"[tiab] OR "hemi-hepat*"[tiab] OR "trisectorectom*"[tiab] OR "bisegmentectom*"[tiab] OR "bi-segmentectom*"[tiab] OR "Hepatectomy"[tiab] OR "Hepatectomy"[Mesh] OR "Carcinoma, Hepatocellular/surgery"[Mesh] OR "Liver Neoplasms/surgery"[Mesh] OR "Liver Diseases/surgery"[Mesh] |
| # 2 | (liver[tiab] OR hepat*[tiab]) AND (surg*[tiab] OR resect*[tiab] OR lobectomy[tiab]) |
| # 3 | #1 OR #2 |
| # 4 | "high volume hospital*"[tiab] OR "low volume hospital*"[tiab] OR "high volume center*"[tiab] OR "high volume centre*"[tiab] OR "low volume center*"[tiab] OR "low volume centre*"[tiab]OR "hospital volume*"[tiab] OR "hospital size*"[tiab] OR "clinic size*"[tiab] OR "center volume*"[tiab] OR "centre volume*"[tiab] OR "high volume center"[tiab] OR "high volume centre"[tiab] OR "center size*"[tiab] OR "centre size*"[tiab] OR "patient volume*"[tiab] OR "provider volume*"[tiab] OR "surgical volume*"[tiab] OR "procedure volume*"[tiab] OR "procedural volume*"[tiab] OR "facility volume*"[tiab] OR "treatment volume*"[tiab] OR "Hospitals, High-Volume"[Mesh] OR "Hospitals, Low-Volume"[Mesh] |
| # 5 | ("hospital centrali*"[tiab] OR "hospital decentrali*"[tiab] OR "regionalization"[tiab] OR "regionalisation"[tiab]) AND volume[tiab] |
| # 6 | "surgery caseload*"[tiab] OR "surgery workload*"[tiab] OR "surgical caseload*"[tiab] OR "surgical workload*"[tiab] OR "surgeon caseload*"[tiab] OR "surgeon workload*"[tiab] OR "surgeon experience*"[tiab] OR "surgeon performance"[tiab] |
| # 7 | ("Surgeons"[Mesh] OR surgeon*[tiab] OR "Treatment Outcome"[Mesh] OR "Patient Outcome Assessment"[Mesh]) AND (volume[tiab] OR caseload[tiab] OR workload[tiab]) |
| # 8 | "volume outcome*"[tiab] |
| # 9 | #4 OR #5 OR #6 OR #7 OR #8 |
| # 10 | #3 AND #9 |
| # 11 | #10 NOT ("Conference Abstract" [Publication type] OR "Comment" [Publication Type] OR "Letter" [Publication Type] OR "Editorial" [Publication Type]) |

| **Cochrane Library:** | |
| --- | --- |
| #1 | MeSH descriptor: [Hepatectomy] explode all trees |
| #2 | MeSH descriptor: [Carcinoma, Hepatocellular] explode all trees |
| #3 | (segmentectom* OR hemi?hepat* OR trisectorectom* OR bi?segmentectom* OR Hepatectomy):ti,ab,kw |
| #4 | ((liver OR hepat*) NEAR/3 (surg* OR resect* OR lobectomy)):ti,ab,kw |
| #5 | #1 OR #2 OR #3 OR #4 |
| #6 | MeSH descriptor: [Hospitals, High-Volume] explode all trees |
| #7 | MeSH descriptor: [Hospitals, Low-Volume] explode all trees |
| #8 | (“high volume hospital*” OR “low volume hospital*” OR “high volume center*” OR “high volume centre*” OR “low-volume center*” OR “low volume centre*” OR “hospital volume*” OR “hospital size*” OR “clinic size*” OR "center volume*" OR "centre volume*" OR "high-volume center" OR "high volume centre" OR "center size*" OR "centre size*" OR "patient volume*" OR "provider volume*" OR "surgical volume*" OR "procedure volume*” OR "procedural volume*” OR "facility volume*” OR "treatment volume*”):ti,ab,kw |
| #9 | (surgeon* AND (volume* OR workload OR caseload)):ti,ab,kw |
| #10 | (volume* NEAR/3 outcome*):ti,ab,kw |
| #11 | (centrali* OR decentrali* OR regionali?ation):ti,ab,kw |
| #12 | MeSH descriptor: [Surgeons] explode all trees |
| #13 | MeSH descriptor: [Patient Outcome Assessment] explode all trees |
| #14 | MeSH descriptor: [Treatment Outcome] explode all trees |
| #15 | #11 OR #12 OR #13 OR #14 |
| #16 | (volume* OR workload OR caseload):ti,ab,kw |
| #17 | #15 AND #16 |
| #18 | #6 OR #7 OR #8 OR #9 OR #10 OR #17 |
| #19 | #5 AND #18 in Cochrane Reviews, Cochrane Protocols and Trials |

| **EMBASE:** | |
| --- | --- |
| # 1 | exp liver resection/ |
| # 2 | exp liver disease/su [Surgery] |
| # 3 | exp liver metastasis/su [Surgery] |
| # 4 | exp liver cell carcinoma/su [Surgery] |
| # 5 | (segmentectom* or hemihepat* or "hemi-hepat*" or trisectorectom* or bisegmentectom* or "bi-segmentectom*" or Hepatectomy).ab,kf,kw,ti. |
| # 6 | (surg* or resect* or lobectomy).ab,kf,kw,ti. |
| # 7 | (liver or hepat*).ab,kf,kw,ti. |
| # 8 | #6 and #7 |
| # 9 | #1 or #2 or #3 or #4 or #5 or #8 |
| # 10 | volume/ or high volume surgeon/ or surgeon volume/ or low volume hospital/ or surgical volume/ or high volume hospital/ or patient volume/ or hospital volume/ or low volume surgeon/ |
| # 11 | ("high volume hospital*" or "low volume hospital*" or "high volume center*" or "high volume centre*" or "low volume center*" or "low volume centre*" or "hospital volume*" or "hospital size*" or "clinic size*" or "center volume*" or "centre volume*" or "high volume center" or "high volume centre" or "center size*" or "centre size*" or "patient volume*" or "provider volume*" or "surgical volume*" or "procedure volume*" or "procedural volume*" or "facility volume*" or "treatment volume*").ab,kf,kw,ti. |
| # 12 | #10 or #11 |
| # 13 | regionalization/ |
| # 14 | exp centralization/ |
| # 15 | ("hospital centrali*" or "hospital decentrali*" or regionali?ation).ab,kf,kw,ti. |
| # 16 | volume*.ab,kf,kw,ti. |
| # 17 | #13 or #14 or #15 |
| # 18 | #16 and #17 |
| # 19 | ("surgery caseload*" or "surgery workload*" or "surgical caseload*" or "surgical workload*" or "surgeon caseload*" or "surgeon workload*" or "surgeon experience*" or "surgeon performance").ab,kf,kw,ti. |
| # 20 | exp surgeon/ or surgeon*.ab,kf,kw,ti. |
| # 21 | exp clinical outcome/ |
| # 22 | exp patient-reported outcome/ |
| # 23 | exp outcome assessment/ |
| # 24 | #21 or #22 or #23 or #20 |
| # 25 | (volume* or caseload or workload).ab,kf,kw,ti. |
| # 26 | #24 and #25 |
| # 27 | (volume* adj3 outcome*).ab,kf,kw,ti. |
| # 28 | #12 or #18 or #19 or #26 or #27 |
| # 29 | #9 and #28 |
| # 30 | limit #29 to "remove medline records" |
| # 31 | #30 not (Conference Abstract or Editorial or Letter).pt. |

| **CENTRAL:** | |
| --- | --- |
| #1 | MeSH descriptor: [Hepatectomy] explode all trees |
| #2 | MeSH descriptor: [Carcinoma, Hepatocellular] explode all trees |
| #3 | (segmentectom* OR hemi?hepat* OR trisectorectom* OR bi?segmentectom* OR Hepatectomy):ti,ab,kw |
| #4 | ((liver OR hepat*) NEAR/3 (surg* OR resect* OR lobectomy)):ti,ab,kw |
| #5 | #1 OR #2 OR #3 OR #4 |
| #6 | MeSH descriptor: [Hospitals, High-Volume] explode all trees |
| #7 | MeSH descriptor: [Hospitals, Low-Volume] explode all trees |
| #8 | (“high volume hospital*” OR “low volume hospital*” OR “high volume center*” OR “high volume centre*” OR “low-volume center*” OR “low volume centre*” OR “hospital volume*” OR “hospital size*” OR “clinic size*” OR "center volume*" OR "centre volume*" OR "high-volume center" OR "high volume centre" OR "center size*" OR "centre size*" OR "patient volume*" OR "provider volume*" OR "surgical volume*" OR "procedure volume*” OR "procedural volume*” OR "facility volume*” OR "treatment volume*”):ti,ab,kw |
| #9 | (surgeon* AND (volume* OR workload OR caseload)):ti,ab,kw |
| #10 | (volume* NEAR/3 outcome*):ti,ab,kw |
| #11 | (centrali* OR decentrali* OR regionali?ation):ti,ab,kw |
| #12 | MeSH descriptor: [Surgeons] explode all trees |
| #13 | MeSH descriptor: [Patient Outcome Assessment] explode all trees |
| #14 | MeSH descriptor: [Treatment Outcome] explode all trees |
| #15 | #11 OR #12 OR #13 OR #14 |
| #16 | (volume* OR workload OR caseload):ti,ab,kw |
| #17 | #15 AND #16 |
| #18 | #6 OR #7 OR #8 OR #9 OR #10 OR #17 |
| #19 | #5 AND #18 in Cochrane Reviews, Cochrane Protocols, Trials |

| **Study registries:** | |
| --- | --- |
| **ICTRP** | ("liver resection" OR hepatectomy OR surgery OR resection OR surgeon OR hospital OR provider OR liver OR institution OR center OR centre OR multicentre OR multicenter) AND (volume OR outcome OR experience OR results OR caseload OR costs OR cost) |
| **Clinicaltrials.gov** | EXPAND[Concept] ( "hospital volume" OR "volume hospital" OR "centre volume" OR "volume centre" OR "center volume" OR "volume center" OR "clinic size" OR "centre size" OR "center size" OR "patient volume" OR "provider volume" OR "surgical volume" OR "procedure volume" OR "procedural volume" OR "facility volume" OR "volume facility" OR "treatment volume" OR "Surgery caseload" OR "surgery workload" OR "surgeon workload" OR "surgeon experience" OR "surgeon skills" OR "experienced surgeon" OR "surgical caseload" OR "surgeon performance" OR "performance surgeon" OR "volume outcome" ) AND AREA[ConditionSearch] ( hepatic OR liver OR Hepatocellular OR hepatic metastasis OR Hepatic OR Liver AND ( Neoplasms OR Diseases OR Cancer ) ) AND AREA[InterventionSearch] ( Liver surgery and liver resection OR surgery OR Resection OR Hepatectomy OR hepatic surgery OR hepatic resection ) |
| **German Clinical Trials Register** | Liver AND resection AND volume |

# Appendix 2: Excluded full-texts

Wrong intervention (including from records identified from systematic reviews): (28)

| Suzuki, H. et al. (2011) ‘Nationwide survey and establishment of a clinical database for gastrointestinal surgery in Japan: Targeting integration of a cancer registration system and improving the outcome of cancer treatment’, Cancer Sci, 102(1), pp. 226–30. |
| --- |
| Nuzzo, G. (2012) ‘Improvement in Perioperative and Long-term Outcome After Surgical Treatment of Hilar Cholangiocarcinoma: Results of an Italian Multicenter Analysis of 440 Patients’, Archives of Surgery, 147(1), p. 26. |
| Lau, K. et al. (2014) ‘The effect of a regional hepatopancreaticobiliary surgical program on clinical volume, quality of cancer care, and outcomes in the Veterans Affairs system’, JAMA Surg, 149(11), pp. 1153–61. |
| Schneider, E.B. et al. (2014) ‘Hospital volume and patient outcomes in hepato-pancreatico-biliary surgery: is assessing differences in mortality enough?’, J Gastrointest Surg, 18(12), pp. 2105–15. |
| Yamashita, Y. et al. (2014) ‘Trends in surgical results of hepatic resection for hepatocellular carcinoma: 1,000 consecutive cases over 20 years in a single institution’, Am J Surg, 207(6), pp. 890–6. |
| van der Geest, L.G. et al. (2015) ‘Survival in relation to hospital type after resection or sorafenib treatment for hepatocellular carcinoma in The Netherlands’, Clin Res Hepatol Gastroenterol, 39(6), pp. 725–35. |
| Mokdad, A.A. et al. (2016) ‘Hospital Volume and Survival After Hepatocellular Carcinoma Diagnosis’, Am J Gastroenterol, 111(7), pp. 967–75. |
| Lagergren, J., Mattsson, F. and Lagergren, P. (2017) ‘Prognosis following cancer surgery during holiday periods’, Int J Cancer, 141(10), pp. 1971–1980. |
| Altieri, M.S. et al. (2018) ‘Academic status does not affect outcome following complex hepato-pancreato-biliary procedures’, Surg Endosc, 32(5), pp. 2355–2364. |
| Haneuse, S. et al. (2018) ‘Assessment of Between-Hospital Variation in Readmission and Mortality After Cancer Surgical Procedures’, JAMA Netw Open, 1(6), p. e183038. |
| Sanaiha, Y. et al. (2018) ‘Incidence and trends of cardiac complications in major abdominal surgery’, Surgery, 164(3), pp. 539–545. |
| Varley, P.R. et al. (2018) ‘Dissemination of Minimally Invasive Liver Resection for Primary Malignancy: Reevaluating Effectiveness’, Ann Surg Oncol, 25(3), pp. 808–817. |
| Wasif, N. et al. (2018) ‘Does Improved Mortality at Low- and Medium-Volume Hospitals Lead to Attenuation of the Volume to Outcomes Relationship for Major Visceral Surgery?’, J Am Coll Surg, 227(1), pp. 85-93.e9. |
| Wasif, N. et al. (2019) ‘Contemporary Improvements in Postoperative Mortality After Major Cancer Surgery are Associated with Weakening of the Volume-Outcome Association’, Ann Surg Oncol, 26(8), pp. 2348–2356. |
| Chen, Q. et al. (2019) ‘Procedure-Specific Volume and Nurse-to-Patient Ratio: Implications for Failure to Rescue Patients Following Liver Surgery’, World J Surg, 43(3), pp. 910–919. |
| Gottlieb-Vedi, E. et al. (2019) ‘Annual hospital volume of surgery for gastrointestinal cancer in relation to prognosis’, Eur J Surg Oncol, 45(10), pp. 1839–1846. |
| Kommalapati, A. et al. (2019) ‘Association between treatment facility volume, therapy types and overall survival in patients with intrahepatic cholangiocarcinoma’, HPB (Oxford), 21(3), pp. 379–386. |
| Mehta, R. et al. (2020) ‘Influence of hospital teaching status on the chance to achieve a textbook outcome after hepatopancreatic surgery for cancer among Medicare beneficiaries’, Surgery, 168(1), pp. 92–100. |
| Olthof, P.B. et al. (2020) ‘Volume–outcome relationship of liver surgery: a nationwide analysis’, British Journal of Surgery, 107(7), pp. 917–926.g |
| Brauer, D.G. et al. (2021) ‘Care Fragmentation and Mortality in Readmission after Surgery for Hepatopancreatobiliary and Gastric Cancer: A Patient-Level and Hospital-Level Analysis of the Healthcare Cost and Utilization Project Administrative Database’, J Am Coll Surg, 232(6), pp. 921-932.e12. |
| Ball, C.G. (2022) ‘Hepatobiliary and pancreatic hemorrhage: Technical tools and tricks’, Surg Open Sci, 7, pp. 22–25. |
| Endo, I. et al. (2021) ‘Mortality, morbidity, and failure to rescue in hepatopancreatoduodenectomy: An analysis of patients registered in the National Clinical Database in Japan’, J Hepatobiliary Pancreat Sci, 28(4), pp. 305–316. |
| de Geus, S.W. et al. (2022) ‘A Rising Tide Lifts All Boats: Impact of Combined Volume of Complex Cancer Operations on Surgical Outcomes in a Low-Volume Setting’, Journal of the American College of Surgeons, 234(6), pp. 981–988. |
| de Geus, S.W. et al. (2021) ‘Combined Hepatopancreaticobiliary Volume and Hepatectomy Outcomes in Hepatocellular Carcinoma Patients at Low-Volume Liver Centers’, J Am Coll Surg, 232(6), pp. 864–871. |
| Hyer, J.M. et al. (2021) ‘A higher hospital case mix index increases the odds of achieving a textbook outcome after hepatopancreatic surgery in the Medicare population’, Surgery, 170(5), pp. 1525–1531. |
| Mehta, R., Tsilimigras, D.I. and Pawlik, T.M. (2021) ‘Assessment of Magnet status and Textbook Outcomes among medicare beneficiaries undergoing hepato-pancreatic surgery for cancer’, J Surg Oncol, 124(3), pp. 334–342. |
| Mueller, M. et al. (2021) ‘Perihilar Cholangiocarcinoma - Novel Benchmark Values for Surgical and Oncological Outcomes From 24 Expert Centers’, Ann Surg, 274(5), pp. 780–788. |
| Wanis, K.N. et al. (2021) ‘Variation in complications and mortality following ALPPS at early-adopting centers’, HPB (Oxford), 23(1), pp. 46–55. |

Wrong Context: (12)

| Nygård, I.E. et al. (2012) ‘Mortality and survival rates after elective hepatic surgery in a low-volume centre are comparable to those of high-volume centres’, ISRN Surg, 2012, p. 783932. |
| --- |
| Hyder, O. et al. (2013) ‘Impact of hospital teaching status on length of stay and mortality among patients undergoing complex hepatopancreaticobiliary surgery in the USA’, J Gastrointest Surg, 17(12), pp. 2114–22. |
| Kluger, M.D. et al. (2013) ‘The learning curve in laparoscopic major liver resection’, J Hepatobiliary Pancreat Sci, 20(2), pp. 131–6. |
| Atallah, C. et al. (2021) ‘Are academic hospitals better at treating metastatic colorectal cancer?’, Surgery, 169(2), pp. 248–256. |
| Booth, C.M. et al. (2016) ‘Surgical resection and peri-operative chemotherapy for colorectal cancer liver metastases: A population-based study’, Eur J Surg Oncol, 42(2), pp. 281–7. |
| Beal, E.W. et al. (2019) ‘Index versus Non-index Readmission After Hepato-Pancreato-Biliary Surgery: Where Do Patients Go to Be Readmitted?’, J Gastrointest Surg, 23(4), pp. 702–711. |
| Cappelle, M. et al. (2020) ‘A multicenter cohort analysis of laparoscopic hepatic caudate lobe resection’, Langenbecks Arch Surg, 405(2), pp. 181–189. |
| Diaz, A. et al. (2021) ‘Association of social vulnerability with the use of high-volume and Magnet recognition hospitals for hepatopancreatic cancer surgery’, Surgery, 170(2), pp. 571–578. |
| Joshi, H.M. et al. (2016) ‘Objective assessment of trainee operative experience in a tertiary hepatobiliary unit’, Eur J Surg Oncol, 42(10), pp. 1548–51. |
| Ihemelandu, C. et al. (2016) ‘Multimorbidity and access to major cancer surgery at high-volume hospitals in a regionalized era’, Am J Surg, 211(4), pp. 697–702. |
| Ibuki, S. et al. (2022) ‘Short-term Outcomes of “Difficult” Laparoscopic Liver Resection at Specialized Centers: Report From INSTALL (International Survey on Technical Aspects of Laparoscopic Liver Resection)-2 on 4478 Patients’, Ann Surg, 275(5), pp. 940–946. |
| Navarro, J.G. et al. (2021) ‘Major Laparoscopic Versus Open Resection for Hepatocellular Carcinoma: A Propensity Score-Matched Analysis Based on Surgeons’ Learning Curve’, Ann Surg Oncol, 28(1), pp. 447–458. |

Single hospital/surgeon only: (4)

| Schwartz, G.S. et al. (2011) ‘Morbidity and mortality after hepatic and pancreatic resections: results from one surgeon at a low-volume urban hospital over thirty years’, Am J Surg, 201(4), pp. 438–44. |
| --- |
| Goh, B.K.P. et al. (2018) ‘Critical appraisal of the impact of individual surgeon experience on the outcomes of laparoscopic liver resection in the modern era: collective experience of multiple surgeons at a single institution with 324 consecutive cases’, Surg Endosc, 32(4), pp. 1802–1811. |
| Goh, B.K. et al. (2021) ‘Evolution and trends in the adoption of laparoscopic liver resection in Singapore: Analysis of 300 cases’, Ann Acad Med Singap, 50(10), pp. 742–750. |
| Ehnstrom, S.R., Siu, A.M. and Maldini, G. (2022) ‘Hepatopancreaticobiliary Surgical Outcomes at a Community Hospital’, Hawaii J Health Soc Welf, 81(11), pp. 309–315. |

Publication type: (6)

| Amato, L. et al. (2017) ‘Volume and health outcomes: evidence from systematic reviews and from evaluation of Italian hospital data’, Epidemiol Prev, 41(5), pp. 1–128. |
| --- |
| Richardson, A.J. et al. (2013) ‘The volume effect in liver surgery–a systematic review and meta-analysis’, J Gastrointest Surg, 17(11), pp. 1984–96. |
| Franken, L.C. et al. (2019) ‘Morbidity and mortality after major liver resection in patients with perihilar cholangiocarcinoma: A systematic review and meta-analysis’, Surgery, 165(5), pp. 918–928. |
| Saulle, R. et al. (2019) ‘The combined effect of surgeon and hospital volume on health outcomes: a systematic review’, Clin Ter, 170(2), pp. e148–e161. |
| Franchi, E., Donadon, M. and Torzilli, G. (2020) ‘Effects of volume on outcome in hepatobiliary surgery: a review with guidelines proposal’, Glob Health Med, 2(5), pp. 292–297. |
| Köpp, F., Ardelt, M. and Settmacher, U. (2021) ‘[Comparison of the outcome of laparoscopic liver resection from high-volume centres with low-volume centres]’, Chirurg, 92(9), p. 848. |

Missing data/not extractable: (6)

| Colavita, P.D. et al. (2014) ‘Regionalization and outcomes of hepato-pancreato-biliary cancer surgery in USA’, J Gastrointest Surg, 18(3), pp. 532–41. |
| --- |
| Ravaioli, M. et al. (2014) ‘A partnership model between high- and low-volume hospitals to improve results in hepatobiliary pancreatic surgery’, Ann Surg, 260(5), pp. 871–5; discussion 875-7. |
| Idrees, J.J. et al. (2017) ‘Cost of Major Complications After Liver Resection in the United States: Are High-volume Centers Cost-effective?’, Ann Surg, 269(3), pp. 503–510. |
| Otsubo, T. et al. (2017) ‘Safety-related outcomes of the Japanese Society of Hepato-Biliary-Pancreatic Surgery board certification system for expert surgeons’, J Hepatobiliary Pancreat Sci, 24(5), pp. 252–261. |
| Tebé, C. et al. (2017) ‘Towards the centralization of digestive oncologic surgery: changes in activity, techniques and outcome’, Rev Esp Enferm Dig, 109(9), pp. 634–642. |
| Uhlig, J. et al. (2019) ‘Hepatocellular carcinoma: Impact of academic setting and hospital volume on patient survival’, Surg Oncol, 31, pp. 111–118. |

Published before 2010: (35)

| Bilimoria, K.Y. et al. (2008) ‘Directing surgical quality improvement initiatives: comparison of perioperative mortality and long-term survival for cancer surgery’, J Clin Oncol, 26(28), pp. 4626–33. |
| --- |
| Birkmeyer, J.D. et al. (2006) ‘Volume and process of care in high-risk cancer surgery’, Cancer, 106(11), pp. 2476–81. |
| Carroll, Jr., J.E. et al. (2009) ‘In-hospital mortality after resection of biliary tract cancer in the United States’, HPB (Oxford), 12(1), pp. 62–7. |
| Chen, T.M. et al. (2008) ‘Management and patient survival in hepatocellular carcinoma: does the physician’s level of experience matter?’, J Gastroenterol Hepatol, 23(7), pp. e179-88. |
| Csikesz, N.G. et al. (2008) ‘Surgical specialization and operative mortality in hepato-pancreatico-biliary (HPB) surgery’, J Gastrointest Surg, 12(9), pp. 1534–9. |
| Dimick, J.B., Cowan, Jr., J.A., et al. (2003) ‘Hepatic resection in the United States: indications, outcomes, and hospital procedural volumes from a nationally representative database’, Arch Surg, 138(2), pp. 185–91. |
| Dimick, J.B., Pronovost, P.J., et al. (2003) ‘Postoperative complication rates after hepatic resection in Maryland hospitals’, Arch Surg, 138(1), pp. 41–6. |
| Dimick, J.B., Cowan, Jr., J.A., et al. (2004) ‘Hospital teaching status and outcomes of complex surgical procedures in the United States’, Arch Surg, 139(2), pp. 137–41. |
| Dimick, J.B., Wainess, R.M., et al. (2004) ‘National trends in the use and outcomes of hepatic resection’, J Am Coll Surg, 199(1), pp. 31–8. |
| Dimick, J.B., Pronovost, P.J. and Lipsett, P.A. (2002) ‘The effect of ICU physician staffing and hospital volume on outcomes after hepatic resection’, Journal of Intensive Care Medicine, 17(1), pp. 41–47. |
| Dixon, E. et al. (2007) ‘Mortality following liver resection in US Medicare patients: does the presence of a liver transplant program affect outcome?’, J Surg Oncol, 95(3), pp. 194–200. |
| Dixon, E. et al. (2009) ‘Population-based review of the outcomes following hepatic resection in a Canadian health region’, Can J Surg, 52(1), pp. 12–7. |
| Eppsteiner, R.W. et al. (2008) ‘High volume and outcome after liver resection: surgeon or center?’, J Gastrointest Surg, 12(10), pp. 1709–16; discussion 1716. |
| Fong, Y. et al. (2005) ‘Long-term survival is superior after resection for cancer in high-volume centers’, Ann Surg, 242(4), pp. 540–4; discussion 544-7. |
| Garcea, G. et al. (2009) ‘A systematic review of the impact of volume of hepatic surgery on patient outcome’, Surgery, 145(5), pp. 467–75. |
| Gasper, W.J. et al. (2009) ‘Has recognition of the relationship between mortality rates and hospital volume for major cancer surgery in California made a difference?: A follow-up analysis of another decade’, Ann Surg, 250(3), pp. 472–83. |
| Gumbs, A.A. and Gayet, B. (2008) ‘Totally laparoscopic central hepatectomy’, J Gastrointest Surg, 12(7), p. 1153. |
| Hollenbeck, B.K. et al. (2007) ‘Volume-Based Referral for Cancer Surgery: Informing the Debate’, Journal of Clinical Oncology, 25(1), pp. 91–96. |
| Li, C.H. et al. (2003) ‘Risk factors associated with intra-operative major blood loss in patients with hepatocellular carcinoma who underwent hepatic resection’, J Chin Med Assoc, 66(11), pp. 669–75. |
| Lin, H.C. et al. (2006) ‘Hospital volume and inpatient mortality after cancer-related gastrointestinal resections: the experience of an Asian country’, Ann Surg Oncol, 13(9), pp. 1182–8. |
| Lin, H.C. and Lin, C.C. (2009) ‘Surgeon volume is predictive of 5-year survival in patients with hepatocellular carcinoma after resection: a population-based study’, J Gastrointest Surg, 13(12), pp. 2284–91. |
| Lordan, J.T. et al. (2009) ‘Operative mortality, blood loss and the use of Pringle manoeuvres in 526 consecutive liver resections’, Ann R Coll Surg Engl, 91(7), pp. 578–82. |
| Lorenzo, C.S. et al. (2005) ‘Factors affecting outcome in liver resection’, HPB (Oxford), 7(3), pp. 226–30. |
| McColl, R.J. et al. (2008) ‘Recent trends of hepatic resection in Canada: 1995-2004’, J Gastrointest Surg, 12(11), pp. 1839–46; discussion 1846. |
| McKay, A. et al. (2008) ‘Impact of surgeon training on outcomes after resective hepatic surgery’, Ann Surg Oncol, 15(5), pp. 1348–55. |
| Metreveli, R.E. et al. (2005) ‘Hepatic resection at a major community-based teaching hospital can result in good outcome’, Ann Surg Oncol, 12(2), pp. 133–7. |
| Nathan, H. et al. (2009) ‘The volume-outcomes effect in hepato-pancreato-biliary surgery: hospital versus surgeon contributions and specificity of the relationship’, J Am Coll Surg, 208(4), pp. 528–38. |
| Nguyen, G.C. et al. (2009) ‘Volumes of liver transplant and partial hepatectomy procedures are independently associated with lower postoperative mortality following resection for hepatocellular carcinoma’, Liver Transpl, 15(7), pp. 776–81. |
| Pal, N. et al. (2008) ‘Volume and outcome for major upper GI surgery in England’, J Gastrointest Surg, 12(2), pp. 353–7. |
| Scarborough, J.E. et al. (2008) ‘Regionalization of hepatic resections is associated with increasing disparities among some patient populations in use of high-volume providers’, J Am Coll Surg, 207(6), pp. 831–8. |
| Shah, S.A. et al. (2007) ‘Survival after liver resection for metastatic colorectal carcinoma in a large population’, J Am Coll Surg, 205(5), pp. 676–83. |
| Simunovic, M. et al. (2006) ‘Influence of hospital characteristics on operative death and survival of patients after major cancer surgery in Ontario’, Can J Surg, 49(4), pp. 251–8. |
| Skipworth, R.J. et al. (2009) ‘The relationship between hospital volume and post-operative mortality rates for upper gastrointestinal cancer resections: Scotland 1982-2003’, Eur J Surg Oncol, 36(2), pp. 141–7. |
| Wang, X. et al. (2007) ‘Predictors of survival after hepatic resection among patients with colorectal liver metastasis’, Br J Cancer, 97(12), pp. 1606–12. |
| Yeung, Y.P., Hui, J. and Yip, W.C. (2008) ‘Hepatectomy for hepatocellular carcinoma in a community hospital: the importance of surgeon procedural volume in operative outcomes’, Hepatogastroenterology, 55(82), pp. 647–52. |

# Appendix 3: Details of surgery types, inclusion criteria and risk adjustment

| **First author and year of publication** | **Surgery types** | **Extent n (%)** | **Inclusion criteria** | **Exclusion criteria** | **Risk adjustment** |
| --- | --- | --- | --- | --- | --- |
| Ardito 2020 [65] | Major hepatectomy (≥3 segments) | 422^a^ (21.8) | - | Liver transplantation, diagnosis of combined primary neoplasms | Age >70, cirrhosis, extent of resection, surgery type |
|  | Minor hepatectomy (<3 segments) | 1513^a^ (78.2) |  |  |  |
| Beal 2019 [78] | Wedge/segmental resection | 7354 (59.9) | STORE codes: 20-59, 65-66 / ICD-O-3 8170; C22.0 | Fibrolamellar HCC and/or metastatic disease | Age, CCI, cN stage, ct, education, income, insurance, race, residence area, gender |
|  | Hemihepatectomy | 4003 (32.6) |  |  |  |
|  | Extended hepatectomy | 909 (7.5) |  |  |  |
| Buettner 2016 [80] | Lobectomy | 1978 (39.0) | ICD-9-CM: 50.3, 50.22./ 155.0; 199.7 | Patient records with missing information on hospital and surgeon identifier | Age, emergency admission, facility type, hospital/surgeon volume, insurance, gender, surgery type, year of treatment |
|  | Partial hepatectomy | 3097 (61.0) |  |  |  |
| Chang 2014 [63] | Major: lobectomy or more | 3370 (25.6) | - | - | Age, cirrhosis, comorbidity, indication, pre-and post-operative cirrhosis-related complication, residence area, gender, socioeconomic status, surgery type, urbanization level of residence |
|  | Minor: < lobectomy | 9789 (74.4) |  |  |  |
| Chapman 2017 [82] | CCCP: |  | STORE codes: 21-22, 26, 50, 59-60 / ICD-O-3 8170-8174; 8180 | Local tumor destruction using photodynamic therapy, fulguration, laser, percutaneous ethanol injection, radiofrequency ablation, cryoablation, liver transplantation, extrahepatic bile duct resection, unknown surgery type; patients with missing facility type, and vital status | AFP, age, AJCC stage, CCI, chemotherapy, cirrhosis, education, facility type, grade, hospital volume, income, insurance, race, radiotherapy, residence area, gender, surgery type, tumor size, year of diagnosis |
|  | Wedge/segmental | 1941 (50.2) |  |  |  |
|  | Lobectomy | 1461 (37.8) |  |  |  |
|  | Extended lobectomy | 287 (7.4) |  |  |  |
|  | Hepatectomy | 181 (4.7) |  |  |  |
|  | ACP: |  |  |  |  |
|  | Wedge/segmental | 4371 (52.0) |  |  |  |
|  | Lobectomy | 2848 (33.9) |  |  |  |
|  | Extended lobectomy | 640 (7.6) |  |  |  |
|  | Hepatectomy | 545 (6.5) |  |  |  |
| Chiu 2015 [52] | Lobectomy | - | ICD-9-CM: 50.3, 50.22 / ICD-9CM 155.xx | Secondary and unspecified malignant neoplasm (ICD-9-CM 196.XX-199.XX), malignant neoplasm of intrahepatic bile ducts (ICD-9-CM code 155.1), malignant neoplasm of liver, not specified as primary or secondary (ICD-9-CM code 155.2) | Age, CCI, cirrhosis, comorbidities, gender, hospital/surgeon volume |
|  | Partial hepatectomy |  |  |  |  |
| Dhar 2019 [72] | Lobectomy | 6476 (100) | ICD-9-CM: 50.3 | Patients with missing data regarding surgeon and center volume; patients younger than 18 years | Comorbidities, complications, hospital/surgeon volume, severity of illness |
| Diggs 2021 [67] | Major hepatectomy ( ≥4 Couinaud liver segments) | 4263 | ICD-O-3 C22.0; 8170-8175; 8160 | Nonoperative cases; resections less than major resections; resections for other malignancies | CCI, crowfly, education, income, insurance, race |
| Eguia 2021 [79] | Lobectomy | 2383 (23.3) | ICD-9-CM: 50.3, 50.22, 54.51, 54.21, 17.41, 17.42, 17.43, 17.49 | <18 years of age; patients with conversion from laparoscopic to open; emergency surgery; liver transplantation; total hepatectomies; robotic procedures | Age, CCI, gender, insurance type, pathology type |
|  | Partial hepatectomy | 7858 (76.7) |  |  |  |
| El Amrani 2019 [69] | Lobectomy | - | CCAM codes: HLFA003-011, HLFA017-020, HLFC002-004, HLFC032, HLFC037; HLEA001-002 / ICD-10 C22.0-4; C22.7; C22.9; C78.7; C24.8; C24.9 | Patients with incorrect patient identification and outside of France; <18 years | Age, CCI, random effect term, gender |
|  | Partial hepatectomy |  |  |  |  |
| Endo 2023 [86] | Minor hepatectomy | 2395 (72.0) | STORE codes: 20-26, 30, 36-38, 50-52, 59, 65, 66 / ICD-O-3 8170 | Patients with stage IV disease, without short- and long-term data, missing data on pathological or clinical staging groups (AJCC) | Age, CCI, facility type, insurance, meld score, presence of fibrosis, race, gender, surgery type, tumor stage and size, year of diagnosis |
|  | Major hepatectomy | 911 (28.0) |  |  |  |
| Farges 2012 [88] | Minor hepatectomies (defined as a single-wedge resection) | 7468 (33.5) | CCAM codes: HLFA003-007, HLFA009-011, HLFA017-020, HLFC002-004, HLFC027, HLFC032, HLFC037 | Ablation; unroofing of simple or parasitic cysts, cystectomy, and necrosectomy had distinct codes and were not considered as liver resections; < 18 years, emergency admissions, cases of hepatobiliary trauma, liver resections corresponding to graft size-reductions, graft donors | Age, associated procedures, comorbidities, hospital structure, indication, palliative surgery, gender, surgery type |
|  | Major hepatectomies (defined as the resection of 3 or more segments) | 7018 (31.5) |  |  |  |
|  | Intermediate hepatectomies (all remaining liver resections) | 7789 (35.0) |  |  |  |
| Filmann 2019 [9] | Segment resection | 11980 | OPS codes: 5-500, 5-502.0-6, 5-501 | - | Not adjusted |
|  | Hepatectomy left | 5570 |  |  |  |
|  | Hepatectomy right | 9026 |  |  |  |
|  | Extended resections | 3885 |  |  |  |
|  | Bisegmentectomy | 4938 |  |  |  |
|  | Other segmentectomies | 7864 |  |  |  |
| Gani 2017 [54] | Lobectomy | 9063 (32.6) | ICD-9-CM: 50.3, 50.22 | Patients undergoing concomitant surgery, emergency surgery | Age, CCI, complications, hospital location, hospital region, hospital teaching status, income quartile, insurance, race, gender, surgery type |
|  | Partial hepatectomy | 18748 (67.4) |  |  |  |
| Gani 2016 [55] | Major hepatectomy 4789 (33.5) | 4789 (33.5) | ICD-9-CM: 50.3, 50.22 | Emergency surgery; <18 years | Age, CCI, income, indication, insurance, gender, surgery type, use of mis |
|  | Partial lobectomy 9507 (66.5) | 9507 (66.5) |  |  |  |
| Görgec 2021 [59] | **Minor:** | Minor ^a^: | - | Preoperatively planned hand-assisted laparoscopy; non-formal resections (i.e. in the case of cyst fenestration/deroofing, biopsies, diagnostic laparoscopy); living donor liver resection was indicated; emergency surgery | Not adjusted |
|  | Wedge/non-anatomical | 735 (30.3) |  |  |  |
|  | Segmentectomy | 195 (8.0) |  |  |  |
|  | Bisgementectomy | 445 (18.4) |  |  |  |
|  | **Technically major:** | Technically major ^a^: |  |  |  |
|  | Wedge/non-anatomical | 439 (18.1) |  |  |  |
|  | Segmentectomy | 106 (4.4) |  |  |  |
|  | Bisgementectomy | 129 (5.3) |  |  |  |
|  | Anatomically major: | Anatomically major ^a^: |  |  |  |
|  | Trisegmentecotmy | 33 (1.4) |  |  |  |
|  | Hemihepatectomy | 333 (13.7) |  |  |  |
|  | Other major hepatectomy | 9 (0.4) |  |  |  |
| Hashimoto 2017 [70] | Lobectomy | - | ICD-9-CM: 50.3, 50.22 | Trauma or recipient (i.e. transplant) hepatectomy | Age, CCI, complications, hepatic necrosis, insurance, need for biliaryenteric reconstruction, presence of hepatic malignancy (primary vs. secondary tumor), race, year of surgery |
|  | Wedge hepatectomy |  |  |  |  |
| Hoerger 2023 [89] | Major hepatectomy (Lobectomy; Extended Lobectomy) | 7528 (42.2) ^a^ | STORE codes: 20-59 | Transplantation, ablation or unclear; patients with unknown 90-day mortality, 30-day readmission, facility type, education level, income level, and unknown insurance status | Age, CCI, education, facility distance, income, insurance, race, gender, year of diagnosis |
|  | Minor hepatectomy (Wedge resection) | 10305 (57.8)^a^ |  |  |  |
| Hunger 2019 [73] | Single segment resection | 1752 | OPS-Codes 5-502.0-5 / ICD-9 CM C78.7, C18.0 | OPS code: 5-502.6 | Age (5-year groups), admission source, clustering, ECI, patient characteristics, gender |
|  | Left hepatectomy | 514 |  |  |  |
|  | Right hepatectomy | 1533 |  |  |  |
|  | Right tresectionectomy | 408 |  |  |  |
|  | Resection of other segment combination | 1319 |  |  |  |
|  | Left lateral sectionectomy | 730 |  |  |  |
| Idrees 2018 [51] | Lobectomy | 29697 ^a^ (30.1) | ICD-9-CM: 50.3, 50.22 | Patients aged <18 years, emergency surgery, transplantation, total hepatectomies | Age, bed size, cci, clustering at the hospital level, hospital charactersticis (area, hospital type), race, gender |
|  | Partial resection | 66409^a^ (69.1) |  |  |  |
| Kohn 2010 [74] | Major hepatectomy | 5298 | ICD-9-CM: 50.3 or ‘greater’ | Minor hepatectomy or wedge resection (ICD-9 codes 50.2, 50.22, 50.29), liver transplantation (ICD-9 codes 50.4 and 50.5) | CCI, surgery year |
| Krautz 2020 [2] | **Major hepatectomies:** | Major: | OPS-Codes 5-502.1-6 | Atypical resections; age <20; cases with procedure codes for post-mortem hepatectomy, liver transplantation, liver graft resection (OPS 5-503 5-504); | Age, comorbidities, indication, gender, surgery type, year of surgery |
|  | Trisectionectomy (TS) | 13987 (45.0)^a^ |  |  |  |
|  | Hemihepatectomy (HH) | 3286 (10.6)^a^ |  |  |  |
|  | Minor hepatectomies: | Minor: |  |  |  |
|  | Multiple segmental resection (MSR) | 8318 (26.7) |  |  |  |
|  | Bisegmentectomy (BS) | 5523 (17.8) |  |  |  |
| Lee 2019 [87] | Partial hepatectomy/segemental resection | 804 (35.6)^a^ | STORE codes for ‘liver cancer sites’ and ‘intrahepatic bile ducts’ | Liver transplantations | Age, CCI, commission on cancer (coc) facility type, distance to hospital, hospital volume, insurance, margin status, race, receipt of adjuvant chemotherapy, gender |
|  | Hepatic lobectomy | 742 (32.9)^a^ |  |  |  |
|  | Extended hepatic lobectomy | 318 (14.1) |  |  |  |
|  | Hepatectomy not otherwise specified | 392 (17.4) |  |  |  |
| Lu 2014 [53] | **Major hepatectomies:** | - | ICD-9-CM: 50.3, 50.22 / ICD-9-CM 155.xx | Secondary and unspecified malignant neoplasm (ICD-9-CM codes 196.XX–199.XX), malignant neoplasm of intrahepatic bile ducts (ICD-9-CM code 155.1), or malignant neoplasm of liver, not specified as primary or secondary (ICD-9-CM code 155.2) | Age, CCI, diagnosis, gender, hospital type, surgeon specialty, surgery type |
|  | Lobectomy |  |  |  |  |
|  | Wedge resection |  |  |  |  |
| Magnin 2023 [62] | Laparoscopic minor (≤2 segments) | 6988 (17.8) | - | <18 years; surgery for infectious or traumatic liver diseases; visceral organ resection during the same procedure (liver resection in combination with resection of colorectal primary tumors) | Age, CCI, comorbidities, indication (malignant versus benign), gender, surgery type, surgical approach (laparotomy versus laparoscopy) |
|  | Laparoscopic major (≥ 3 segments) | 704 (1.8) |  |  |  |
|  | Open minor (≤2 segments) | 20 515 (52.2) |  |  |  |
|  | Open major (≥ 3 segments) | 12 390 (33.2) |  |  |  |
|  | Extensive resection | 2143 (5.5) |  |  |  |
| McColl 2013 [61] | Lobectomy | 293 (28.4)^a^ | Before 04/2002: ICD-9-CM: 50.3, 50.22; After 04/2002: CCI* 1.0A.87.^^ |  | Age, CCI, complications, diagnosis, selective admission, severity of disease, gender, surgeon specialty, surgeon volume, surgery type, surgery year |
|  | Partial resection | 740 (71.6)^a^ |  |  |  |
| Miura 2016 [64] | MOS hepatectomies (more than one segment), excluding the lateral segment | 14970 | - - | MOS hepatectomies performed by laparoscopic procedures were excluded from this study; excluding lateral segment | Not adjusted |
| Munir 2023 [90] | Wedge/segmental resection | 2051 (38.3) | STORE codes: 20-60, 66 / ICD-O-3 8160 | Clinical metastatic disease; liver transplantation; surgery at other sites; patients with missing data for the individual parameters making up the composite TOO metric | Age, analytic stage, CCI, education, facility affiliation, facility location, hospital volume, insurance, race, gender, surgical approach, travel distance, tumor size, year of diagnosis |
|  | Lobectomy | 1696 (31.6) |  |  |  |
|  | Extended Lobectomy | 789 (14.7) |  |  |  |
|  | Partial hepatectomy | 489 (9.1) |  |  |  |
|  | Not otherwise specified | 334 (6.2) |  |  |  |
| Okinaga 2018 [56] | Limited resection | 8653 (31.9) | - ICD-10 C22.0 | <20 years | Age, clustering, comorbidities, hospital type, gender, surgery type |
|  | Segmentecotmy | 9474 (35.0) |  |  |  |
|  | Hemihepatecotmy | 4829 (17.8) |  |  |  |
|  | Extended hemihepatectomy | 3008 (11.1) |  |  |  |
|  | Laparoscopic hepatectomy | 1130 (4.2) |  |  |  |
| Sahara 2020 [76] | Lobectomy | 1491 (20.8) | ICD-9 CM: 50.3, 50.22, 17.49, 54.51. 54.21, 17.43, 17.42, 17.41, 17.49, 54.21 | <65 years; ablation; trauma patients, liver transplantation; patients with missing information for hospital and surgeon identifier | Age, caseload, CCI, cirrhosis, hospital type, indication, race, gender, surgery type |
|  | Partial Hepatectomy | 5678 (79.2) |  |  |  |
| Sato 2012 [57] | Partial hepatectomy | 2163 (41.0)^a^ | K695-(1-5) / ICD-10 C22.0 | Patients who underwent two or more types of the included procedures, TAE for controlling tumor bleeding during emergency hospitalization | Age, comorbidities, surgery type |
|  | Hepatic segmentectomy | 1921 (36.5)^a^ |  |  |  |
|  | Hepatic lobectomy | 869 (16.5)^a^ |  |  |  |
|  | Extended hepatic lobectomy and extended hepatic lobectomy with revascularization procedure | 317 (6.0)^a^ |  |  |  |
| Shaw 2013 [71] | Lobectomy |  | ICD-9-CM: 50.3, 50.22 | Surgeons performing <5 surgeries/year | Hospital volume, primary diagnosis, severity of illness, surgeon specialty, surgeon volume |
|  | Partial hepatectomy |  |  |  |  |
| Siegel 2021 [68] | Wedge/segementecomy | 3872(56.4) |  | Different primary tumor; no diagnosis or treatment data from the reporting facility; missing follow up data; metastatic disease at presentation; unknown stage; wrong travel distance criteria | Age, CCI, education, hospital location, income, gender, surgery type, urban/rural status |
|  | Lobecotmy | 2414 (35.2) |  |  |  |
|  | Extended lobectomy | 574 (8.4) |  |  |  |
| Spolverato 2014 [83] | “At least a hemihepatectomy” | 3960 (40.1) | ICD-9-CM: 50.3, 50.4, 50.22 / ICD-9CM 155.0, 197.7 |  | Age, comorbidities, emergency surgery, race, gender, surgery type |
|  | “Less than a hemihepatectomy” | 5914 (59.9) |  |  |  |
| Sutton 2016 [75] | Lobectomy | 4163 | ICD-9-CM: 50.3 | <18 years | Center volume, discharge status, severity of illness |
| Tsilimigras 2021 [77] | Lobectomy and Partial hepatectomy: |  | ICD-9-CM: 50.3, 50.22, 17.4, 17.41, 17.42, 17.43, 17.49 | Not enrolled in Medicare Part A or B at the time of surgery, patients with additional payments from a health maintenance organization (HMO), patients with payments from a primary payer | Admission type, age, CCI, cirrhosis, hospital type, hospital volume, indication (benign or malignant), race/ethnicity, region, gender, surgery type, year of surgery |
|  | Minimally invasive | 12110 (91.6) |  |  |  |
|  | Open surgery | 1112 (8.4) |  |  |  |
| van der Poel 2019 [60] | **Minor MILS:** | Minor MILS | - | Insufficient data; non-surgical interventions (e.g. fenestration/deroofing of cysts, biopsies, diagnostic laparoscopy); emergency surgery | Not adjusted |
|  | Wedge/non-anatomical resection | 331 (36.1)^a^ |  |  |  |
|  | Segmentectomy | 78 (8.5)^a^ |  |  |  |
|  | Bisegmentectomy | 247 (27.0)^a^ |  |  |  |
|  | **Technically major MILS:** | Technically major MILS |  |  |  |
|  | Wedge/non-anatomical resection | 141 (15.4)^a^ |  |  |  |
|  | Segmentectomy | 21 (2.3)^a^ |  |  |  |
|  | Bisegmentectomy | 34 (3.8)^a^ |  |  |  |
|  | **Anatomically major MILS:** | Anatomically major MILS |  |  |  |
|  | Trisegmentectomy | 11 (1.2)^a^ |  |  |  |
|  | Hemihepatectomy | 47 (5.1)^a^ |  |  |  |
|  | Other major hepatectomy | 5 (0.5)^a^ |  |  |  |
| Viganò 2020 [66] | Left lateral sectionectomy | 280 (12.7)^a^ | - | Unclear procedure undertaken and/or postoperative outcome, laparoscopic pericystectomy; complex and major resections | Not adjusted |
|  | Limited resections of anterolateral segments | 748 (33.8)^a^ |  |  |  |
|  | Segmentectomy of anterolateral segments | 357 (16.2)^a^ |  |  |  |
|  | Bisegementectomy of anterolateral segments | 87 (3.9)^a^ |  |  |  |
|  | Resection of posterosuperior segments | 460 (20.8)^a^ |  |  |  |
|  | Right anterior sectionectomy | 10 (0.5)^a^ |  |  |  |
|  | Right posterior sectionectomy | 41 (1.9)^a^ |  |  |  |
|  | Major hepatectomy | 227 (10.3)^a^ |  |  |  |
| Yasunaga 2012 [58] | Limited resection | 7582 (42.0) | Limited resection (K6951), segmentectomy (K6952), lobectomy (K6953), extended lobectomy without vascular or biliary reconstruction (K6954), or extended lobectomy with reconstruction (K6955) | Living-donor liver transplantation | Age, CCI, diagnosis, hospital type, gender, surgery type |
|  | Segmentectomy | 5422 (30.0) |  |  |  |
|  | Lobectomy | 3099 (17.2) |  |  |  |
|  | Extended lobectomy without reconstruction | 1527 (8.5) |  |  |  |
|  | Extended lobectomy with reconstruction | 416 (2.3) |  |  |  |

^a^, self-calculated

# Appendix 4: Volume definitions by volume type

| **Hospital volume** | | | | | | |
| --- | --- | --- | --- | --- | --- | --- |
| **First author and year of publication** | **Patients (N)** | **Case volume category** | | **Hospital volume / year** | **Patients / category** | **Units / category** |
| Ardito 2020 [65] | 1935 | - | Low | 21-50 | 162 | 5 |
|  |  |  | Intermediate^b^ | 51-100 / 21-100^b^ | 830 / 674 ^b^ | 10 |
|  |  |  | High | >100 / >100^b^ | 943/ 674 ^b^ | 3 |
| Beal 2019 [78] | 12226 | Quartiles / continuous per 10 cases | First | ≤1 | 3040 | ^-^ |
|  |  |  | Second | 1.1- 4 | 3067 |  |
|  |  |  | Third | 4.1-12.5 | 3091 |  |
|  |  |  | Fourth | ≥12.5 | 3068 |  |
| Chapman 2017 [82] | 12757 | - | Low | <10 | 7106 | 1003 |
|  |  |  | High | ≥10 | 5637 | 57 |
| Diggs 2021 [67] | 4263 | - | Low | <15 | 3342 | 590 |
|  |  |  | High | ≥15 | 921 | 17 |
| Eguia 2021 [79] | 10239 | - | Low | <53 | 2662 | - |
|  |  |  | Moderate | 53-119 | 2523 |  |
|  |  |  | High | 120-314 | 2625 |  |
|  |  |  | Very high | >314 | 2431 |  |
| El Amrani 2019 [69] | 28763 | - | Low | <76 | 16928 | 680^a^ |
|  |  |  | High | ≥76 | 11835 | 35^a^ |
| Endo 2023^1^ [86] | 3268 | - | Low | <50 | 2782 (85.1) | - |
|  |  |  | High | ≥50 | 486 (14.9) |  |
| Farges 2012 [88] | 22275 | Unnamed categories | ≤ 5 | ≤5 | 2176 | 13 |
|  |  |  | 6-10 | 6-10 | 2226 |  |
|  |  |  | 11-25 | 11-25 | 3282 |  |
|  |  |  | 26-50 | 26-50 | 2772 |  |
|  |  |  | 51-100 | 51-100 | 4618 |  |
|  |  |  | >100 | >100 | 7151 |  |
| Filmann 2019 [9] | 40034 | - | Very Low | 1-4 | 0.17 – 4 | 460 |
|  |  |  | Low | 5-13 | 4- 13.2 | 80 |
|  |  |  | Medium | 14-44 | 13.2 - 43.5 | 26 |
|  |  |  | High | 45-71 | 43.5 - 71.5 | 9 |
|  |  |  | Very High | 72-171 | 71.5 – 171 | 5 |
| Gani 2017 [54] | 27813 | - | Low | 1-4 | 1472 (5.4) | 952 (43.1) |
|  |  |  | Intermediate | 5-14 | 2030 (7.5) | 590 (26.7) |
|  |  |  | High | ≥15 | 24309 (87.1) | 665 (30.1) |
| Gani 2016 [55] | 14296 | - | Low | ≤12 | 4341 | 1573 |
|  |  |  | Intermediate | 12-45 | 4608 | 218 |
|  |  |  | High | ≥45 | 5347 | 66 |
| Görgec 2021 [59] | 2425 | - | Low | <50 | 1540 | 20 |
|  |  |  | High | ≥50 | 885 | 3 |
| van der Poel 2019 [60] | 916 | - | Low | <20 | 137 | 2014-2016: 19, 15, 14; 2014-2016: 1, 5, 6 |
|  |  |  | High | ≥20 | 123 |  |
| Hoerger 2023 [89] | 17833 | Percentiles | 10^th^ | ≥2 | - | 310 |
|  |  |  | 25^th^ | ≥7 | - | 130 |
|  |  |  | 50^th^ | ≥26 | - | 52 |
|  |  |  | 75^th^ | ≥46 | 14206 | 18 |
|  |  |  | 90^th^ | ≥59 | 3627 | 5 |
| Hunger 2019 [73] | 5900 | Quartiles | - | 1-2 | 1412 | - |
|  |  |  |  | 3-5 | 1533 |  |
|  |  |  |  | 6-12 | 1357 |  |
|  |  |  |  | 13-40 | 1578 |  |
| Idrees 2018 [51] | 96107 | - | Low | 1-50 | 52472 (55) | - |
|  |  |  | Medium | 51-149 | 29931 (31) |  |
|  |  |  | High | >150 | 13704 (14) |  |
| Kohn 2010 [74] | 5298 | - | Continuous | Incremental effect of each hepatectomy | - | - |
| Krautz 2020 [2] | 31114 | - | Very low | 1-10 | 9734 | mean 403.3 |
|  |  |  | Low | 11-20 | 4953 | mean 50.1 |
|  |  |  | Medium | 21-40 | 4648 | mean 24.0 |
|  |  |  | High | 41-100 | 6555 | mean 14.6 |
|  |  |  | Very high | >100 | 5224 | mean 5.4 |
| Lee 2019 [87] | 2256 | - | Low | <11 | - | - |
|  |  |  | High | ≥11 |  |  |
| Magnin 2023 [62] | 39286 | - | Low | ≤25 | 13375 (34.0) | 293 |
|  |  |  | High | >25 | 25911 (66.0) | 43 |
| Miura 2016 [64] | 14970 | - | Categories and  continuous | <10 | 1512 | - |
|  |  |  |  | ≥10 | 13458 |  |
|  |  |  |  | ≥50 | 7040 |  |
|  |  |  |  | ≥100 | 3152 |  |
| Munir 2023 [90] | 5359 | - | Low | <3 | 2120 | - |
|  |  |  | Moderate | 3-7 | 2421 |  |
|  |  |  | High | >7 | 818 |  |
| Okinaga 2018 [56] | 27094 | - | Very low | ≤ 9 | - | - |
|  |  |  | Low | 10-23 |  |  |
|  |  |  | High | 24-51 |  |  |
|  |  |  | Very high | > 52 |  |  |
| Sato 2012 [57] | 5270 | - | Low | ≤ 21 | 1784 | - |
|  |  |  | Intermediate | 20-56 | 1742 |  |
|  |  |  | High | ≥ 57 | 1744 |  |
| Yasunaga 2012 [58] | 18046 | - | Very low | <18 | 4674 | 596 |
|  |  |  | Low | 18-35 | 4396 | 149 |
|  |  |  | High | 36-70 | 4569 | 74 |
|  |  |  | Very high | >70 | 4407 | 36 |
| Siegel 2021^2^ [68] | 4359 | - | Low | <153 | - | - |
|  |  |  | High | >466 |  |  |
| Spolverato 2014 [83] | 9874 | - | Low | <21 | 3950 | 147 |
|  |  |  | Intermediate | 21-43 | 2161 | 28 |
|  |  |  | High | >43 | 3763 | 20 |
| Sutton 2016 [75] | 4163^a^ | - | Low | 1-15 | 1-16 | 1-16 |
|  |  |  | Medium | 16-31 | 17-29 | 17-26 |
|  |  |  | High | 34-58 | 30-86 | 27-78 |
| Tislimigras 2020^5^ [77] | 1112 | - | Average | 1-3 | 421 / 468 / 461 | 77 / 78 / 74 |
|  |  |  | Above average | 4-7 | 453 / 455 / 464 | 20 / 20 / 22 |
|  |  |  | High | ≥8 | 472 / 474 / 496 | 11 / 11 / 13 |
| Viganó 2020 [66] | 2225 | - | Low / low | ≤2^3^ / ≤50^4^ | 849 / 442 | 37 / 23^a^ |
|  |  |  | High / intermediate/high | >2 / >50 | 1376 / 407 | 9 / 23 |

| **Surgeon volume** | | | | | | |
| --- | --- | --- | --- | --- | --- | --- |
| **First author and year of publication** | **Patients (N)** | **Case volume category** | | **Surgeon volume / year** | **Patients / category** | **Units / category** |
| Hashimoto 2017 [70] | 13467 | - | Continuous | - | - | - |
| McColl 2013 [61] | 1033 | - | Low | 1-4 | - | - |
|  |  |  | High | ≥5 |  |  |
| Sahara 2020 [76] | 7169 | - | Low | ≤2 | 3922 | - |
|  |  |  | Medium | 3-5 | 2301 |  |
|  |  |  | High | ≥6 | 946 |  |

| **Hospital and surgeon volume** | | | | | | | |
| --- | --- | --- | --- | --- | --- | --- | --- |
| **First author and year of publication** | **Patients (N)** | **Case volume category** | | **Hospital volume / year** | **Surgeon volume / year** | **Patients / category** | **Units / category** |
| Buettner 2016 [80] | 5075 | - | Low | ≤11 | ≤4 | 1824 / 1722 | 360 / 720 |
|  |  |  | Intermediate | 12-45 | 5-15 | 1568 / 1720 | 38 / 55.7 |
|  |  |  | High | ≥46 | ≥16 | 1683 / 1633 | 10 |
| Chang 2014 [63] | 13159 | - | Low | <245 | <25 | 6294 / 8319 |  |
|  |  |  | High | ≥245 | ≥25 | 6865 / 4840 | 13 / 59 |
| Chiu 2015 [52] (Lu 2014 [53]) | 23107 | - | Low | 1-99 | 1-29 | 12305 / 11818 | - |
|  |  |  | High | ≥100 | ≥30 | 10802 / 11289 |  |
| Dhar 2019 [72] | 6476 | - | Low | 1-18 | 1-4 | - | - |
|  |  |  | Medium | 19-30 | 5-11 |  |  |
|  |  |  | High | >30 | >12 |  |  |
| Shaw 2013 [71] | 8692 | - | Low | <40 | <12 | - | - / 27 |
|  |  |  | Intermediate | 40-100 | 12-38 |  | - / 13 |
|  |  |  | High | >100 | >38 |  | - / 10 |

*H* Hospital, *S* Surgeon; ^a^ self-calculated; ^b^ PSM; ^c^ July-December; ^d^ November-May; ^e^ reported/year, available from digital appendix; ^1^ Cases/6-years:
^2^ Cases/10-years; ^3^ Minimally invasive cases/month; ^4^ Minimally and open surgery cases per year; ^5^ Study included 13222 patients: 13100 open surgery, 1112 MILS

# Appendix 5: Hospital volume level mortality results, stratified by mortality type

| **First author and year of publication** | **Hospital Mortality / FTR** | **Quality** **appraisal** | |
| --- | --- | --- | --- |
|  |  | **ISPOR** (Yes/Partially/No/NA) | **ROBINS-E** (Domain 7†) |
| **Anatomical resections** | | | |
| Hunger 2019 [73] | **Hospital mortality:** (OR): 1-2 vs. 3-5: 0.94^a^ [0.61-1.44]; 6-12: 1.19 ^a^ [0.78-1.84]; 13-40: 1.25 ^a^ [0.82-1.94] | 10/8/3/6 | High Risk |
| Krautz 2020 [2] | **Hospital mortality:** Major hepatectomies: (OR): Very low vs. Low: -; Medium: 0.73^a^ [0.6-0.9]; High: 0.65^a^ [0.5-0.8]; Very high: 0.59^a^ [0.4–0.9]\| **FTR:** (%): Very low: 29.38^d^ [26.7–32.2]; Low : 27.13^d^ [23.9–30.7]; Medium: 28.05^d^ [24.8–31.6]; 41-100: 24.0^d^ [21.7–26.5]; High: 21.38^d^ [19.2–23.8]\| **Hospital mortality:** Minor hepatectomies (OR): Very low vs. Low: -; Medium: 0.73^a^ [0.6-0.9]; High: 0.65^a^  [0.5-0.8]; Very high: 0.59^a^ [0.4–0.9]\| **FTR:** (%): Very low: 17.9^d^ [15.6–20.5]; Low: 15.33^d^ [12.0–19.3]; Medium: 14.0^d^ [10.6–18.1]; High: 17.1^d^ [13.7–21.1]; Very high: 13.54^d^ [10.1–17.7] | 11/3/7/6 | High Risk |
| Kohn 2010 [74] | **Hospital mortality:** (OR): Incremental effect: 0.975^a^ [0.967-0.983]*** | 9/1/11/6 | High Risk |
| **Anatomical and non-anatomical resections separately** | | | |
| Eguia 2021 [79] | **Hospital mortality:** (OR): **Lobectomy:** Low-to-high p<0.05 vs. Very high: 0.47^a^ [0.12 -1.82]\|  **Partial hepatectomy:** Low-to-high p<0.05 vs. Very high: 0.66^a^ [0.16-2.67] | 11/1/9/6 | High Risk |
| Magnin 2023 [62] | **FTR:** (OR): Low vs. High: 0.79^a^ [0.65-0.97]***\| **Hospital mortality:** (OR): Low vs. High: 0.74^a^ [0.58-0.93]*** **FTR:** (OR): **Minor hepatectomy:** Low vs. High: 0.82^a^ [0.67-0.99]***; Hospital mortality: Low vs. High: 0.80^a^ [0.65-0.99]*\| **FTR:** (OR): **Major hepatectomy:** Low vs. High: 0.91^a^ [0.70-1.19]; Hospital mortality: Low vs. High: 0.83^a^ [0.63-1.10] | 8/3/10/6 | High Risk |
| **Anatomical and non-anatomical resections combined** | | | |
| Ardito 2019 [65] | **FTR:** High vs. Low-intermediate: 5.995^a^ [1.961-18.328]**;  (After PSM): High vs. Low-intermediate: 5.069^a^ [1.409–18.232]* | 9/4/8/6 | Low Risk |
| Buettner 2016 [80] | **Postoperative mortality:** (OR): High vs. Low: 2.13^a^ [1.31-3.47]**; Intermediate: 2.00^a^ [1.24-3.21]**\| **FTR:** High vs. Low: 2.15^a^ [1.33–3.48]**; Intermediate: 2.04^a^ [1.25–3.33]** | 12/2/7/6 | High Risk |
| Gani 2016 [55] | **Postoperative mortality:** (%): Low: 9.0^d^ ; Intermediate: 7.6^d^; High: 1.3^d^ *\| **FTR:** Low: 16.6^d^; Intermediate: 24.7^d^; High: 15.1^d^ ** | 11/4/6/6 | High Risk |
| Shaw 2013 [71] | **Hospital Mortality:** (OR): Low vs. Intermediate -; High: 0.44^a^ [0.13-0.56] | 11/4/6/6 | High Risk |
| Gani 2017 [54] | **Postoperative mortality:** (OR): Low vs. Intermediate: 0.78^a^ [0.61-0.99]*; High: 0.68^a^ [0.51-0.92]** | 11/2/8/6 | High Risk |
| Farges 2012 [88] | **Hospital mortality**: ≤ 5 vs. 6-10: 0.89^a^ [0.53–1.49]; 11-25: 0.56^a^ [0.34–0.93]*; 26-50: 0.54^a^ [0.30–0.96]*; 51-100: 0.52^a^ [0.29–0.94]*; > 100: 0.56^a^ [0.30–0.95]* | 10/1/10/6 | High Risk |
| Chiu 2015 [52] | **Hospital mortality^1^:** (%): Low: 3.4^d^ ; High: 1.4^d^  ***\| | 9/2/10/6 | High Risk |
| Sato 2012 [57] | **Hospital mortality:** (OR): High vs. Low: 2.74^a^ [1.74-4.30]***; Intermediate: 1.45^a^ [0.88-2.38] | 8/1/12/6 | High Risk |
| Spolverato 2014 [83] | **Hospital mortality:** (OR): High vs. Low: 1.50^a^ [1.13-1.99]**; Intermediate: 1.73^a^ [1.25-2.39]**\| **FTR:** High vs. Low: 1.40^a^ [1.02-1.93]**; Intermediate: - | 8/1/12/6 | High Risk |
| Idrees 2018 [51] | **FTR:** (OR): Low vs. Medium: 0.95^a^ [0.61-1.48]; High: 0.69^a^ [0.37-1.27] | 7/3/11/6 | High Risk |

| **First author and year of publication** | **30-day Mortality** | **Quality** **appraisal** | |
| --- | --- | --- | --- |
|  |  | **ISPOR** (Yes/Partially/No/NA) | **ROBINS-E** (Domain 7†) |
| **Anatomical resections** | | | |
| None | | | |
| **Anatomical and non-anatomical resections separately** | | | |
| Hoerger 2023 [83] | **Mortality:** (OR): **Major hepatectomy:** (30 day): Low vs. High: 0.58^a^ [0.41–0.75]***\| **Mortality:** (OR): **Any hepatectomy:** (30 days): Low vs. High: 0.55^a^ [0.42–0.73]*** | 11/1/9/6 | Low Risk |
| Yasunaga 2012 [58] | **All-cause mortality:** (OR): (30-day): Very low vs. Low: 0.70^a^ [0.48-1.02]; High: 0.52^a^ [0.34-0.81], p<0.01; Very high: 0.16^a^ [0.09-0.30], p<0.001 | 8/1/12/6 | High Risk |
| **Anatomical and non-anatomical resections combined** | | | |
| Lee 2019 [84] | **Mortality:** (OR): (30-day): Low vs. High: 0.68^a^ [0.40-1.16]\| | 7/4/10/6 | High Risk |
| Chang 2014 [63] | **Mortality:** (OR): (30-day): High vs. Low: 1.50^b^ [1.09–2.07]**\| | 8/3/10/6 | High Risk |

| **First author and year of publication** | **90-day Mortality** | **Quality** **appraisal** | |
| --- | --- | --- | --- |
|  |  | **ISPOR** (Yes/Partially/No/NA) | **ROBINS-E** (Domain 7†) |
| **Anatomical resections** | | | |
| Diggs 2021 [67] | **Mortality:** (90 days): RR: High vs. Low: 1.60^c^ [1.25–2.05]*** | 8/2/11/6 | High Risk |
| **Anatomical and non-anatomical resections separately** | | | |
| Hoerger 2023 [89] | **Mortality:** (OR): **Major hepatectomy:** (90 day): Low vs. High: 0.62^a^ [0.49–0.80]***  **Mortality:** (OR): **Any hepatectomy:** (90 days): Low vs. High: 0.67^a^ [0.52–0.87]** | 11/1/9/6 | Low Risk |
| **Anatomical and non-anatomical resections combined** | | | |
| Ardito 2019 [65] | **Mortality:** (OR): (90-day): High vs. Low: 5.625^a^ [1.050-30.131]*; Intermediate: 7.119^a^ [1.050-30.131]**/ FTR: High vs. Low-intermediate: 5.095^a^ [1.878-13.821]**\| | 9/4/8/6 | Low Risk |
| El Amrani 2019 [69] | **Mortality:** (OR): (90-day): High vs. Low: 1.34^a^ [1.07-1.67]** | 10/2/9/6 | Low Risk |
| Okinaga 2018 [56] | **Mortality:** (OR): (90-day): Very low vs. Low: 0.84^a^ [0.67 - 1.05]; High: 0.60^a^ [0.47 - 0.78]***; Very high: 0.36^a^ [0.27 - 0.49]*** | 9/1/11/6 | Low Risk |
| Beal 2019 [78] | **HR:** (30 to 90 day mortality): Q1 (lowest) vs. Q2: 1.11^b^ [0.90–1.37]; Q3: 0.97^b^ [0.79–1.18]; Q4: 0.69^b^ [0.58–0.82]**\| ***Per 10 cases:*** 0.981^b^ [0.977–0.984]** | 11/0/10/6 | High Risk |
| Farges 2012 [88] | **Mortality:** (OR): (90-day): ≤ 5 vs.6-10: 0.81^a^ [0.56–1.16]; 11-25: 0.50^a^ [0.35–0.72]***;  26-50: 0.58^a^ [0.39–0.86]**; 51-100: 0.51^a^ [0.34–0.78]**; > 100: 0.53^a^ [0.34–0.82]** | 10/1/10/6 | High Risk |
| Chiu 2015 [52] | **Mortality:** (HR): (90-day): Low vs. High: 0.79^b^ [0.698-0.887]***\| | 9/2/10/6 | High Risk |
| Lee 2019 [87] | **Mortality: (90-day):** Low vs. High: 0.68^a^ [0.46-1.01]\| | 7/4/10/6 | High Risk |
| Chang 2014 [63] | **Mortality: (3-months):** High vs. Low: 1.56^b^ [1.30–1.86]***\| | 8/3/10/6 | High Risk |

| **First author and year of publication** | **Intermediate-/long-term Mortality** | **Quality** **appraisal** | |
| --- | --- | --- | --- |
|  |  | **ISPOR** (Yes/Partially/No/NA) | **ROBINS-E** (Domain 7†) |
| **Anatomical resections** | | | |
| None | | | |
| **Anatomical and non-anatomical resections separately** | | | |
| None | | | |
| **Anatomical and non-anatomical resections combined** | | | |
| Beal 2019 [78] | **OS:** (median): Q1 (lowest): 30.4; Q2: median 31.84; Q3: median 37.65; Q4: median 51.7*\| | 11/0/10/6 | High Risk |
| Siegel 2021 [68] | **OS: (HR):** High vs. Low: 0.74^b^ [0.64-0.87]***\| **(excluding 30-day):** High vs. Low: 0.76^b^ [0.65-0.89]***\| **(excluding 90-day):** High vs. Low: 0.75^b^ [0.64-0.88]*** | 9/3/9/6 | High Risk |
| Chiu 2015 [52] | **Mortality: (5-years):** Low vs. High: 0.91^b^ [0.873-0.970]**\| **5-year mortality^1^:** Low: 41.7^d^ ;High: 32.8^d^ ***\|  **OS^1^:** (months): Low: 68.6^e^ (0.6); High: 77.1^e^ (0.7)*** | 9/2/10/6 | High Risk |
| Lee 2019 [87] | **Mortality:** (OR): (1-year): Low vs. High: 0.95^a^ [0.79-1.14] | 7/4/10/6 | High Risk |
| Chang 2014 [63] | **Mortality:** (OR): (1-year): High vs. Low: 1.33^b^ [1.21–1.46]** | 8/3/10/6 | High Risk |
| Endo 2023^2^ [86] | **OS:** (HR): Low vs. High: 0.83^b^ [0.69-0.99]* | 8/1/12/6 | High Risk |
| Chapman 2017^3^ [82] | **Survival:** High vs. Low CCCP: 1.14^b^ [1.05-1.22]***\| High vs. Low ACP: 1.13^b^ [1.06-1.20]*** | 6/3/12/6 | High Risk |

^a^ OR [95% CI]; ^b^ HR [95% CI]; ^c^ RR [95% CI]; ^d^ Rate in % [95% CI]; ^e^ mean (SD); *P value < 0.05; **P value < 0.01; ***P value < 0.001; *FTR* Failure to rescue; *OS* Overall survival; *ACP* Academic cancer programs; *CCCP* Comprehensive community cancer program; ^1^ Outcomes from Lu et al. 2014; ^2^ Minimal invasive cases / 6 years; ^3^ 1-year, 5-year, 10-year survival in digital appendix
Note: † Domain 7 = Risk of bias in selection of reported result; Reference categories were underscored if available

# Appendix 6: Surgeon volume level mortality results, stratified by mortality type

| **First author and year of publication** | **Hospital Mortality / FTR** | **Quality appraisal** | |
| --- | --- | --- | --- |
|  |  | **ISPOR** (Yes/Partially/No/NA) | **ROBINS-E** (Domain 7†) |
| **Anatomical and non-anatomical resections combined** | | | |
| Buettner 2016 [80] | **Postoperative mortality:** (OS): High vs. Low: 3.01^a^ [1.80–5.04]***; Intermediate: 2.56^a^ [1.54–4.26]***\| **FTR:** High vs. Low: 3.42^a^ [1.98–5.93]***; Intermediate: 3.08^a^ [1.77–5.34]*** | 12/2/7/6 | High Risk |
| Shaw 2013 [71] | **Hospital mortality:** (OR): Low vs. Intermediate: -; High: 0.55^a^ [0.33-0.89] | 11/4/6/6 | High Risk |
| Chiu 2015 [52] | **Hospital mortality^1,2^:** (%): Low: 4.2^d^; High: 1.0^d^***\| | 9/2/10/6 | High Risk |

| **First author and year of publication** | **Short-term Mortality** | **Quality appraisal** | |
| --- | --- | --- | --- |
|  |  | **ISPOR** (Yes/Partially/No/NA) | **ROBINS-E** (Domain 7†) |
| **Anatomical and non-anatomical resections combined** | | | |
| Chiu 2015 [52] | **Mortality:** (HR): (90-day): Low vs. High: 0.86^b^ [0.822-0.902]*** | 9/2/10/6 | High Risk |
| Chang 2014 [63] | **Mortality:** (OS): (30-day): High vs. Low: 1.64^b^ [1.12–2.41]*\|  **(3-month):** High vs. Low: 1.62^b^ [1.31–2.00]**\| | 8/3/10/6 | High Risk |
| Tsilimigras 2021 [77] | **Mortality:** (OR): (30-day): Average (minimally invasive only) vs. Above average: -; High: 0.59^a^ [0.45-0.78]\| **(90-day):** Average (minimally invasive only) vs. Above average: -; High: 0.64^a^ [0.51-0.79] | 8/3/10/6 | High Risk |

| **First author and year of publication** | **Mortality outcomes** | **Quality appraisal** | |
| --- | --- | --- | --- |
|  |  | **ISPOR** (Yes/Partially/No/NA) | **ROBINS-E** (Domain 7†) |
| **Anatomical and non-anatomical resections combined** | | | |
| Chiu 2015 [52] | **Mortality:** (HR): **(5-year):** Low vs. High: 0.84^b^ [0.799-0.878]***\| **(5-year)^1,2^:** Low: 43.9^d^; High: 30.2^d^**\|** **OS^1,2^:** (months): Low: 66.9^e^ (0.7); High: 78.5^e^ (0.7)** | 9/2/10/6 | High Risk |
| Chang 2014 [63] | **Mortality:** (OR): (1-year): High vs. Low: 1.33^b^ [1.21–1.46]** | 8/3/10/6 | High Risk |

^a^ OR [95% CI]; ^b^ HR [95% CI]; ^c^ RR [95% CI]; ^d^ Rate in % [95% CI]; ^e^ mean (SD); *P value < 0.05; **P value < 0.01; ***P value < 0.001; *FTR*: Failure to rescue; *OS*: Overall survival; ^1^ Outcomes from Lu et al. 2014; ^2^ Propensity-score matched cohort;
Note: † Domain 7 = Risk of bias in selection of reported result; Reference categories were underscored if available

# Appendix 7: Harvest plots of adjusted results

**
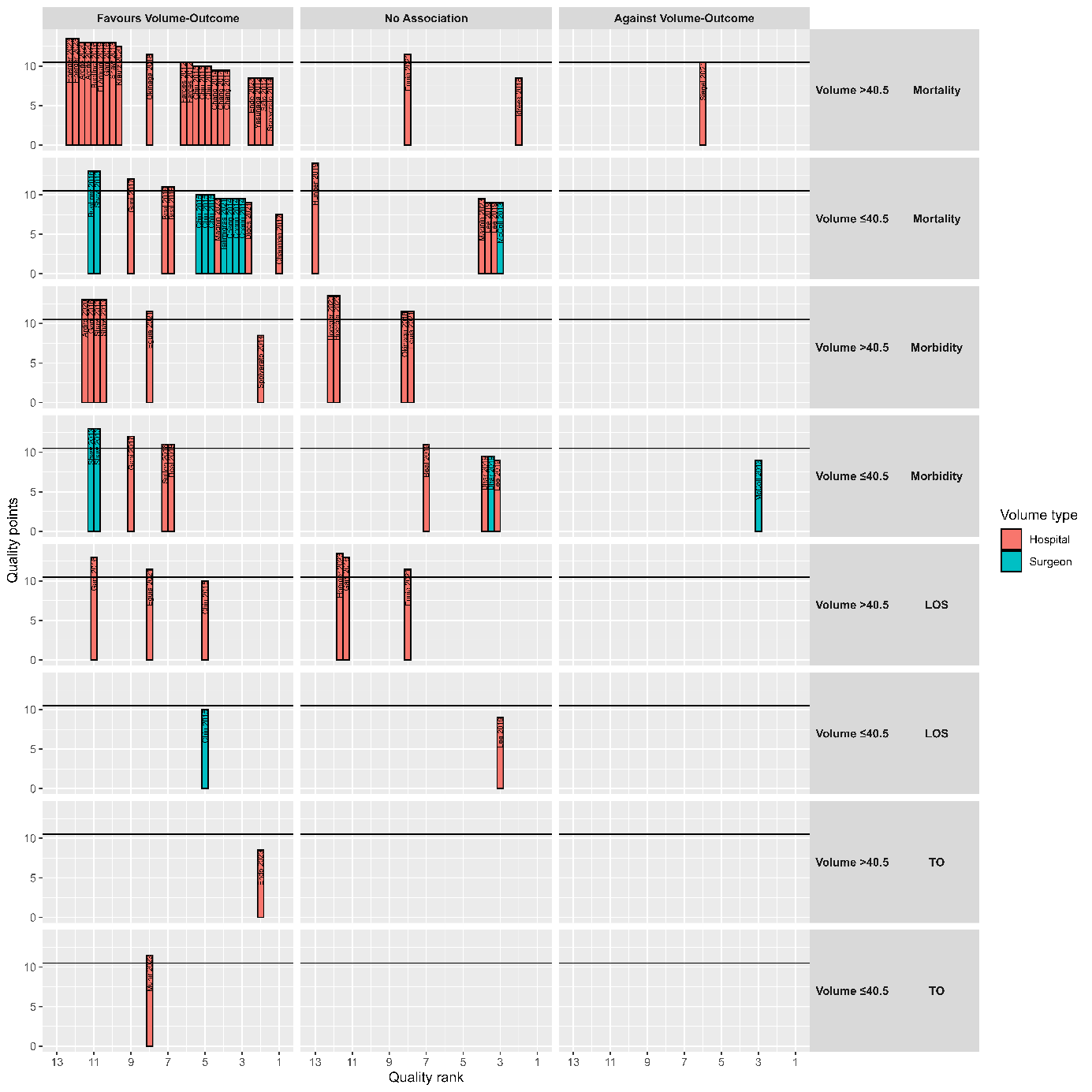
**

This harvest plot is stratified by outcome categories and sub-stratified by their median volume cutoff for “high-volume” definition. Each bar represents one study effect in its respective categories and is assigned to either no effect, an effect in favor of a volume-outcome relationship with outcomes or against an effect in favor of a volume-outcome relationship. The height of the bar corresponds to the quality score, while the horizontal position represents the rank of the study amongst the included studies. The horizontal black line denotes the quality cutoff, similar to the summary table 9. This allows to compare how many studies report effects for each outcome, their nominal quality as well as their relative quality across all strata.

Appendix 8: Harvest plots of adjusted results, sensitivity analysis
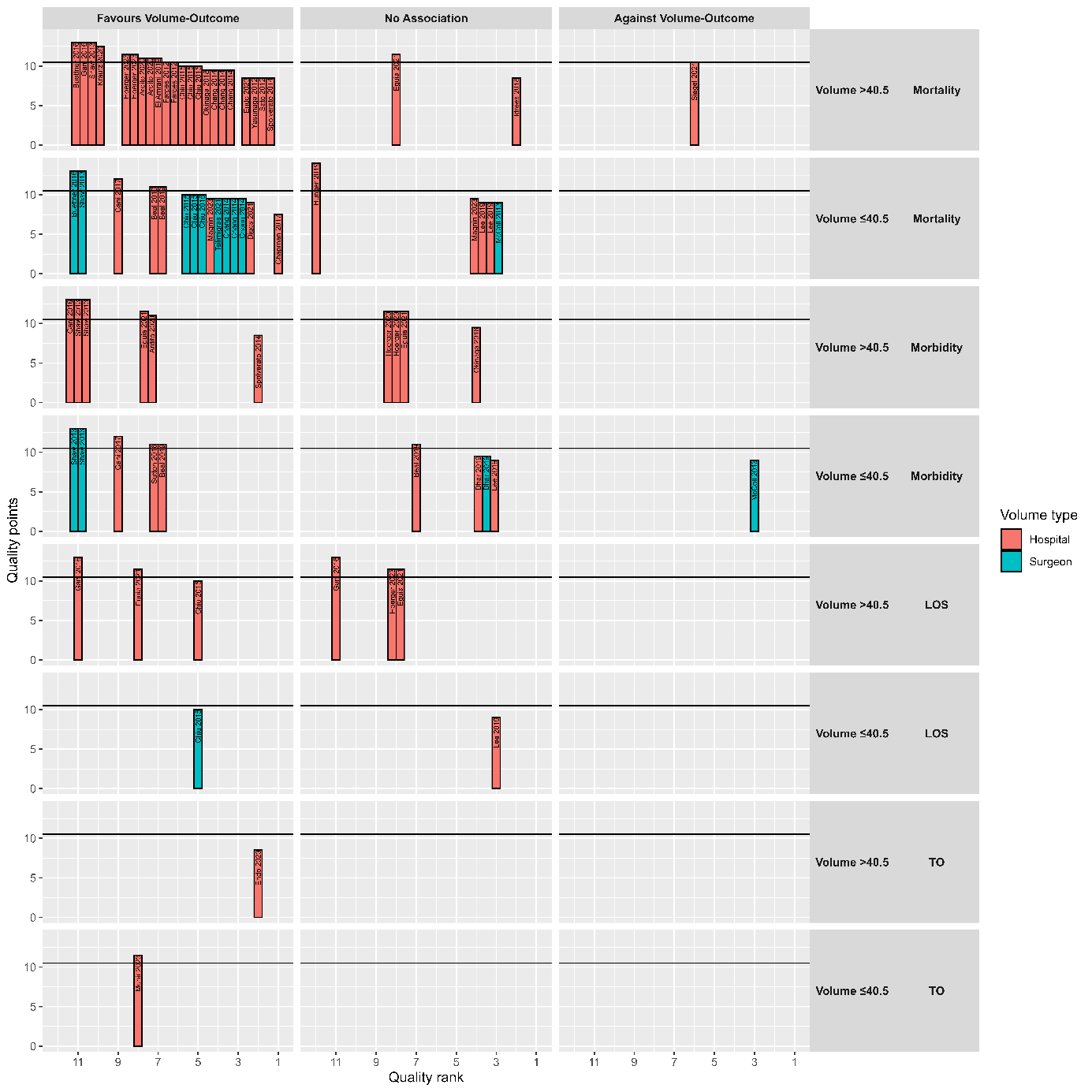


This harvest plot is analogous to appendix 7, but the quality score is modified to omit the ROBINS-E summation as a sensitivity analysis. Quality rank is adapted to this alternative specification.
